# Rumination Out Loud? Linguistic, Neural, and Psychophysiological Correlates of the Think‐Aloud Paradigm

**DOI:** 10.1155/da/8855110

**Published:** 2026-04-23

**Authors:** Isabell Int-Veen, Andreas J. Fallgatter, Ann-Christine Ehlis, David Rosenbaum

**Affiliations:** ^1^ Department of Psychiatry and Psychotherapy, Tübingen Center for Mental Health (TüCMH), University of Tübingen, Tübingen, Germany, uni-tuebingen.de; ^2^ German Center for Mental Health, partner site Tübingen, Tübingen, Germany

**Keywords:** natural language processing, rumination, sentiment analysis, think aloud paradigm, trier social stress test

## Abstract

Stress and rumination are closely linked and contribute to the development and maintenance of mental disorders, yet assessing rumination in an ecologically valid way remains challenging. Conducting the think aloud paradigm (TAP) following the Trier Social Stress Test (TSST) allows for the real‐time evaluation of ruminative responses, providing insights beyond traditional self‐report measures. This study aimed to investigate ruminative responses to the TSST using the TAP, while simultaneously assessing psychological (stress, affect, state rumination), physiological (heart rate), and neural data with functional near‐infrared spectroscopy (fNIRS). For this, a total of 58 healthy participants (mean age 23.47 years (*SD* = 3.87), 63.8% females) completed a 10‐min resting‐state period both before and after the TSST, verbalizing their thoughts. In response to the TSST, we observed significant increases in stress, state rumination, negative affect, heart rate, and cortical oxygenation in all regions of interest except the inferior frontal gyrus (IFG), reflecting a successful stress induction. Although we observed higher stress, state rumination, and negative affect, alongside lower positive affect in high ruminators using questionnaires, linguistic evaluation of the verbalized thought content showed no significant time effects but revealed generally lower sentiment scores for high ruminators and only partly showed differences dependent on trait rumination levels. With respect to neural correlates, we observed prefrontal hypoactivation under stress in medium and high compared to low ruminators. Comparing the results with previous studies, the administration of the TAP following the TSST seems to function as a form of emotion regulation, thereby reducing state rumination. Further studies are required to explore the underlying mechanisms.

## 1. Introduction

Rumination is defined as a perseverative, highly self‐referential, negative, and abstract thinking style with little or no goal and change orientation [1, 2]. Several psychopathologies are associated with ruminative thinking: for example depression, anxiety disorders, eating disorders, and substance use disorders [1], which is why rumination is considered a transdiagnostic mechanism [3–8].

To date, various theoretical frameworks have been proposed to explain ruminative thinking, each emphasizing distinct core aspects and thus implying differing degrees of temporal stability [9]. A prominent example is the influential Response Styles Theory by Nolen‐Hoeksema and Morrow [10], which conceptualizes rumination as a trait. In contrast, other lines of research highlight rumination as a context‐dependent, reactive response to stress [11, 12]. Although the latter perspective has been acknowledged as a valuable addition to existing models [9], research on rumination continues to rely heavily on trait questionnaires such as the Ruminative Response Scale (RRS; [10]), and the Perseverative Thinking Questionnaire (PTQ; [13]). This is even despite the availability of several psychometrically validated state rumination questionnaires: For instance, the Brief State Rumination Inventory (BSRI; [14]), or the Stress‐Reactive State Rumination Questionnaire (SRSRQ; [15]). These are specifically designed to capture situational variability and temporal fluctuations of ruminative thought, allowing for the detection of meaningful within‐person changes over time.

An alternative approach to questionnaires is the real‐time assessment of thought content. This idea was first implemented in the so‐called Articulated Thoughts in Simulated Situations paradigm (ATSS; [16]) where participants hear audio‐recordings of a conversation they are supposed to imagine being part of and later articulate their emotions. Several studies have implemented the ATSS paradigm to investigate psychological processes like emotion regulation (e.g., [17–19]); however, one crucial element of this paradigm is that participants have to imagine being part of the audio‐recording and need to empathize with the situation to respond to the received prompts. That means the ATSS is most probably limited in its ecological validity. Another paradigm is the think aloud paradigm (TAP) which goes back to think‐aloud verbalization trials by Ericsson and Simon [20], where information is verbalized as it is still available in short‐term memory. The application of the TAP has been broadly diversified since then: TAP has been used to understand stress and coping in sports [21–23] or complex processes like decision‐making (e.g., [24, 25]) and problem‐solving (e.g., [26, 27]).

So far, there is only one study that used the TAP in the context of rumination: Raffaelli et al. [28] instructed participants to verbalize everything that came to mind, including internal thoughts, perceptions of external stimuli, and bodily sensations without any prompts. Assessing two samples of undergraduates, participants sat alone in a quiet, normally lit testing room for 10 min. Manually rating the transcripts, the authors found that the TAP is highly ecologically valid and trait brooding was linked to more negative thoughts, which were past‐oriented and self‐focused. Other studies investigating the linguistic features of ruminative thinking used (clinical) interview data and observed similar results: Brockmeyer et al. [29] instructed participants to describe one of the saddest and happiest moments in their lives and found that self‐referent word‐use is associated with brooding. Stade et al. [30] investigated the natural language correlates of perseverative thinking, an umbrella term encompassing worry and rumination [31]. They used clinical interviews from patients with depression, generalized anxiety disorder and healthy controls and found that I‐usage and negative emotion language were significantly associated with perseverative thinking.

Using the TAP in the context of ruminative thinking and stress provides the opportunity to capture the underlying complex cognitive processes in real time, thereby enabling the investigation of the full cognitive sequence, its context dependency, and temporal dynamics, rather than relying solely on participants’ evaluations of their current state. Moreover, TAP is likely to be less susceptible to retrospective biases. Such a time‐dependent, state‐oriented assessment can offer unique insights into the mechanisms underlying rumination and its fluctuations across and following stressful situations. By linking real‐time thought content to physiological and neural responses, this approach may improve our understanding of how rumination contributes to stress‐related psychopathology and inform the development of targeted interventions or preventive strategies by getting to know how rumination unfolds.

In this study, we sought to apply the TAP in a manner analogous to Raffaelli et al. [28], but unlike the authors, who gave no prompts, we aimed to indirectly trigger rumination using the Trier Social Stress Test (TSST; [32]) and implemented the TAP in resting‐states prior to and after the TSST. Previous studies have already found the TSST to be a highly ecologically valid stressor that reliably induces rumination [33–41]. To further gain insight into the neural correlates, we measured cortical oxygenation using functional near‐infrared spectroscopy (fNIRS), which has been found suitable in studies involving TAP (e.g., [42, 43]) and the TSST [35, 38, 39, 44–51]. Previous studies using the TSST have indicated increases in cortical oxygenation between non‐stressful control conditions and the arithmetic task of the TSST while high ruminators and patients with depression show prefrontal hypoactivation, particularly in the right inferior frontal gyrus (IFG) and left dorsolateral prefrontal cortex (DLPFC) [38, 39, 44, 48, 49, 51].

fNIRS was recorded during two control tasks prior to the stress induction and throughout the TSST, but not during the two resting‐state measurements during which the TAP was performed. During these periods, participants were alone, and we aimed to minimize potential risks associated with the laser optodes while ensuring that they felt comfortable verbalizing every thought. Previous studies on the neural underpinnings of rumination have shown increases in (primarily left‐sided) brain regions of the default mode network (DMN) like the left IFG, left precuneus and left anterior cingulate, among others (see, e.g., meta‐analysis by Zhou et al. [52]) but also greater activation and connectivity within the amygdala, medial prefrontal cortex and posterior cingulate cortex (see, e.g., [53–65]). When specifically investigating the neural correlates of state rumination, aberrant functioning of the (left) DLPFC [39, 44, 66], the medial prefrontal cortex and the anterior cingulate cortex [67, 68] and the right IFG has been repeatedly reported [38]. However, due to the variety of applied experimental procedures and neuroimaging methods future studies are urgently needed in order to gain more insight into the underlying neural mechanisms. Very recent findings on verbalized spontaneous thought during a resting‐state fMRI in healthy adult participants revealed rather broad and diffuse activation not only apparent in the DMN [69], which is in line with findings from previous meta‐analysis and studies on functional neuroimaging studies of mind‐wandering and related spontaneous thought processes [70, 71].

By adopting the same experimental design as Rosenbaum et al. [39, 49, 51] including a resting‐state prior to and after the TSST but with the additional implementation of a TAP during the resting‐state, the present study aimed to evaluate the validity of the TAP as a method for assessing stress‐reactive rumination. In line with this objective, we first sought to replicate the analyses conducted by Rosenbaum et al. [49], focusing on psychological and (neuro‐)physiological stress. Specifically, we hypothesized that, following the TSST, high ruminators would exhibit greater increases in state rumination and negative affect compared to low ruminators, whereas we expected no significant group‐by‐time interactions for subjective stress ratings, heart rate, or math performance during the TSST. We also examined math performance, as cortical oxygenation and task performance are closely linked, with increased recruitment of neural resources typically supporting better performance. Based on a previous study with a comparable sample and procedure, we expected an increase in the number of calculations and errors from the non‐stressful control task to the TSST, but we did not anticipate differences between groups [38]. Further, we expected significant increases in cortical oxygenation under stress in the bilateral DLPFC, left IFG and SAC. We expected low ruminators to show increased activation in prefrontal areas, while high ruminators should show reduced activation during the TSST [38, 39, 44, 51], mediating higher state rumination and negative affect.

Secondly, we aimed to explore the ecological validity of the TAP for capturing state rumination. To this end, we examined whether verbalized thoughts during the TAP were modulated by the stress induction—specifically, whether the frequency of negatively valenced words increased as assessed via sentiment analysis, and whether observer‐rated content analysis revealed changes in rumination. We expected high ruminators to show stronger increases in negative thought content and ruminative verbalizations following the TSST, compared to few or no changes in low ruminators.

## 2. Methods

### 2.1. Participants

Participants were informed about the study via the university mailing list. A total of 74 participants were recruited. Participants had to be 18–40 years old, had normal or corrected‐to‐normal vision, and were native or highly proficient speakers of German. They were excluded if they had significant medical, neurological, or mental disorders, acute substance use, pregnancy, or any other factor that could interfere with safe participation or have an impact on the (neuro‐)physiological stress response (for details on the inclusion and exclusion criteria see Supporting Information 1: S1). After excluding 16 participants due to loss of interest (*n* = 8), no longer fulfilling inclusion criteria (*n* = 5) and not completing the tasks (*n* = 3), the final sample comprised *N* = 58 participants (see CONSORT‐diagram in Supporting Information 1: S2). For a sensitivity analysis, as well as post hoc power analysis, showing that the achieved power for all reported effects was ≥0.80, see Supporting Information 1: S3.

The mean age of the sample was 23.47 years (*SD* = 3.87) and 63.8% were female. All participants gave written informed consent. This study was approved by the ethics committee at the University Hospital and University of Tübingen (Project Number 738/2023BO2).

During the preparation of this work the authors used ChatGPT (GPT‐5.2) to assist with language editing and grammar checking. After using this tool, the authors reviewed and edited the content as needed and take full responsibility for the content of the published article.

### 2.2. Procedure

**Figure 1:**
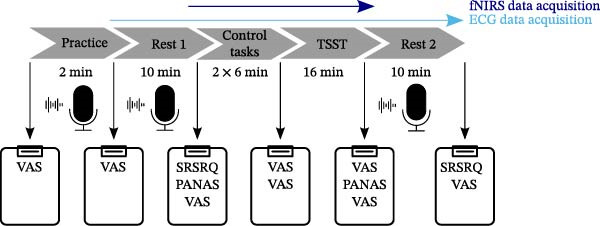
Overview of the study procedure. Microphones illustrate the think aloud task, Questionnaires illustrate the completion of questionnaires. ECG, electrocardiogram; fNIRS, functional near‐infrared spectroscopy; PANAS, positive and negative affect schedule; SRSRQ, stress‐reactive state rumination questionnaire; TSST, trier social stress test; VAS, subjective stress rating using a visual analog scale.

The experimental procedure is illustrated in Figure 1. Data for the study were collected at the University Hospital Tübingen between December 2023 and September 2024.

In the following, we aim to give a short overview of the experimental procedure focusing on the temporal sequence. Later, in separate subsections, we give detailed explanations about the individual components.

After arriving at the laboratory, participants gave written informed consent and completed several questionnaires assessing sociodemographic data, trait rumination using the RRS [72], symptoms of depression using the Beck Depression Inventory II (BDI‐II; [73]), symptoms of social anxiety using the Liebowitz Social Anxiety Scale (LSAS; [74]). Then, participants completed the first of in total 8 stress ratings using Visual Analog Scales (VAS) ranging from 0% to 100% by answering the question “Please tick how stressed you currently feel, including the last 5 minutes.” Psychometric properties and further details of all questionnaires used in this study are reported in Supporting Information 1: S4.

Participants were then introduced to the TAP. To familiarize them with the task, they completed a 2‐min practice phase during which a study nurse remained present to ensure that participants articulated their thoughts clearly into the microphone and fully understood the instructions. After the practice phase, the study nurse left the room and the first 10‐min resting‐state period was conducted, during which participants performed the TAP. Subsequently, the study nurse re‐entered the room and two control tasks were completed. These were followed by the TSST. Finally, a second 10‐min resting‐state period was conducted, during which participants again performed the TAP. Finally, participants were debriefed and received 20 € or 2 h of course credit (Figure 1).

### 2.3. Think Aloud Paradigm

The TAP was performed during a practice phase and then during both resting‐state measurements. Prior to practicing the TAP, participants received a verbal instruction by the study nurse to verbalize all thoughts as they occurred and to speak continuously into a microphone placed on the table in front of them. We used a microphone and popkiller by the t.bone (SC 440 USB‐microphone and MS‐180 Popkiller). Then, participants practiced the TAP for 2 min while a study nurse was present in the room, who ensured that participants verbalized their thoughts continuously and spoke loud enough into the microphone so that transcripts could be automatically generated (see section 2.6 for further information). After 2 min of practice, participants completed a second stress rating using the VAS. The study nurse then provided them with written instructions (see Supporting Information 1: S5) for the upcoming 10‐min resting‐state (rest1), before leaving the room. Participants were asked to “sit comfortably” and to “allow their thoughts to flow freely” and to verbally express any spontaneous thoughts. They were further informed that an audio recording would be made. No other individuals were present in the room during this 10‐min period, allowing participants to speak freely without disturbance. Further, blinds were closed while participants sat in the normally lit testing room in order to minimize external stimulation. Following this first resting‐state, participants responded to questions on another VAS, evaluating the extent to which they had self‐censored during the task (0%–100%). This rating was based on Raffaelli et al. [28] and was intended to assess the extent to which the spoken content truly reflected the participants’ thoughts or whether the participants were attempting to suppress or refrain from verbalizing their actual thoughts. Additionally, current stress, affect using the Positive and Negative Affect Schedule (PANAS; [75]), and state rumination (SRSRQ; [15]) were assessed. Meanwhile, participants were prepared for the fNIRS‐measurement (see Supporting Information 1: S6). After the TSST, subjective stress and affect were assessed before another 10‐min resting‐state followed during which the TAP was performed. Then, another SRSRQ was completed. During practicing and both resting‐states in which the TAP was administered, only electrocardiogram data were recorded (for details, see Supporting Information 1: S7), as participants were alone and we aimed to minimize any potential risks associated with the fNIRS laser optodes.

### 2.4. Control Tasks

Participants completed two control tasks, which served as control conditions for the arithmetic task of the TSST. In the first control task (ctl1), they were instructed to read number series for 40 s following 20 s of rest in 6 trials. Ctl1 was implemented to account for basic visual, attentional, and verbal demands while keeping cognitive load and stress levels low. Afterward, another stress rating was conducted. In the second control task (ctl2), participants were asked to perform mental arithmetic, counting backward in 13‐step increments from different starting numbers for 40 s each, again followed by 20 s of rest and a total of 6 trials. If participants made errors, they were instructed to start over with the initial number. After ctl2, participants gave another stress rating. In addition to accounting for basic visual, attentional, and verbal demands, ctl2 targeted task‐related cognitive processing and error monitoring without imposing time constraints or social evaluation. It should be noted, however, that although these control tasks were designed to be less stressful than the arithmetic task of the TSST, cognitive load and stress are closely intertwined and cannot be entirely separated [76].

### 2.5. TSST

Next, the TSST was conducted. Two interviewers wearing white coats entered the room and sat at a table in front of the participant. The TSST panel consisted of research assistants and included either two females or one male and one female—never two males, due to team composition. Participants were instructed to imagine that they had applied for a job at the University Hospital and that it was the day of the job interview. As part of the interview, they were asked to deliver a speech about their personal strengths and qualifications for the job. Participants were given 5 min to prepare the speech and could take notes. After the preparation period, the TSST panel collected the notes before participants were asked to present their speech. No time limit for the speech was mentioned to the participant. Participants were video‐ and audio‐recorded without prior notice. If a participant stopped speaking before 5 min had elapsed, they were prompted to continue.

After the speech, participants’ stress levels were assessed again, followed by a 6‐min arithmetic task analogous to ctl2 and again comprised a total of six trials with 40 s of calculations followed by 20 s of rest. This time, they were instructed to calculate as quickly and accurately as possible while maintaining eye contact with one of the interviewers, which further increased socio‐evaluative threat. Although the original publication of the TSST does not explicitly mention maintaining eye contact [32], this procedure is specified in official TSST protocols [77]. Mistakes were interrupted by the interviewers, who asked participants to start over. This task structure resulted in a 6‐min task duration, compared to the original 5 min. In addition, whereas the original task requires participants to start from the same number each time, we used different starting numbers for each trial. We observed that in the original paradigm, participants tend to memorize the initial numbers when repeating them across trials. Moreover, for the calculation of event‐related averages in the fNIRS analysis, it is necessary to have distinct trials with intervening pauses to allow the hemodynamic response to recover. During the interview as well as the arithmetic task, the TSST panel remained socially non‐responsive (no nodding or smiling) and neutral. Upon completion of the TSST, the fNIRS probe set was removed.

### 2.6. Transcripts

Transcripts were automatically generated from the recordings and corrected by an independent rater no further involved in the data analysis. Using R, we calculated sentiment scores for each transcript, which quantify the overall emotional valence of the verbalized thoughts, capturing the balance of positive and negative language in participants’ responses.

Scores ranged from –1 (very negative) to +1 (very positive) (for details see Supporting Information 1: S8). In addition to the automated sentiment‐analysis that captures only negative thought content, we conducted an observer‐rated content rating of the resting‐state transcripts to capture further characteristics of rumination. Specifically, the first and last authors independently evaluated the transcripts. Based on prior studies [50, 51], four scales were rated: (1) rehashing bad performance, (2) speculation on negative consequences, (3) focus on negative affect, and (4) reflection. Text segments could be assigned to multiple categories and were rated on a 5‐point Likert scale: 0 = not present to 4 = strongly present. Weighted Cohen’s *κ* ranged between 0.38 and 0.71 (see Supporting Information 1: S9). For the analysis, we averaged the ratings of the two raters.

### 2.7. Data Analysis

**Figure 5:**
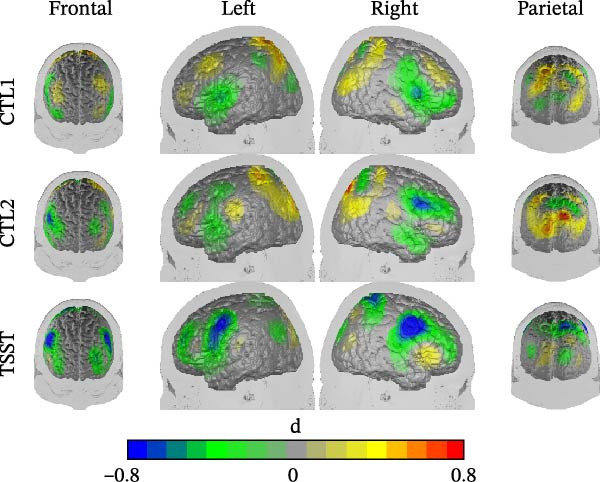
Differences between high and low RRS during control task 1 (CTL1 = reading numbers), control task 2 (CTL2 = performing calculations without social stress) and TSST (arithmetic task of the TSST). Cool colors indicate reduced O_2_Hb‐levels in the high RRS group as compared to the low RRS group; warm colors vice versa. Differences are depicted in Cohen’s *d*.

Data analysis was performed using SPSS Version 28 [78]. Figures were generated using R Version 4.3.1 [79] and the ggplot2 package [80] (Figures 2–4), while brain maps shown in Figure 5 were plotted using MATLAB 2024a. Please note that the analysis of this study was not preregistered.

Figure 2Subjective stress ratings (0%–100%) (A) and heart rates in beats per minute (BPM) (B) dependent on trait rumination group (RRS group). Boxplots illustrate the distribution of the raw data whereby the central line of the boxplots indicates the median and the hinges extend to the 25th and 75th percentiles, respectively. Colored jittered dots represent the individual raw data for each RRS group. The bold dots and connecting line depict the group means.

**Figure:**
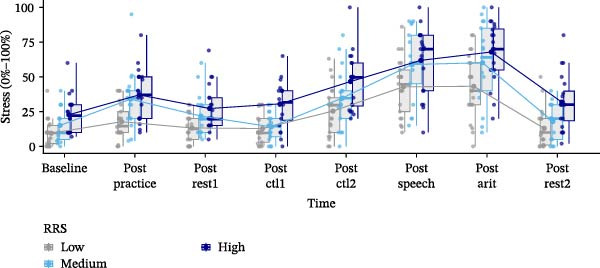
(A)

**Figure:**
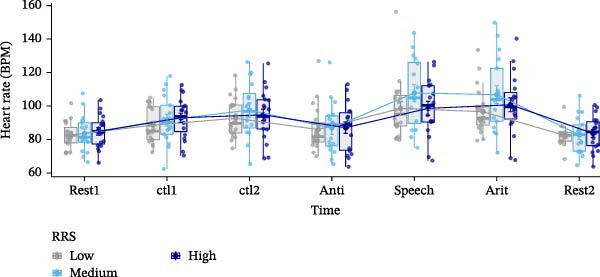
(B)

Figure 3Sentiment scores (A), positive (B), and negative affect ratings (PANAS scores) (C), as well as state rumination (SRSRQ scores) (D) dependent on trait rumination group (RRS group). Boxplots illustrate the distribution of the raw data whereby the central line of the boxplots indicates the median and the hinges extend to the 25th and 75th percentiles, respectively. Colored jittered dots represent the individual raw data for each RRS group. The bold dots and connecting line depict the group means.

**Figure:**
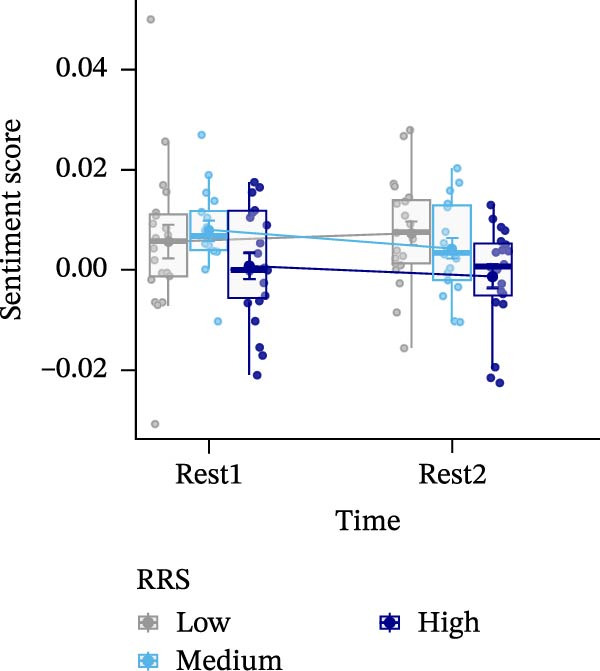
(A)

**Figure:**
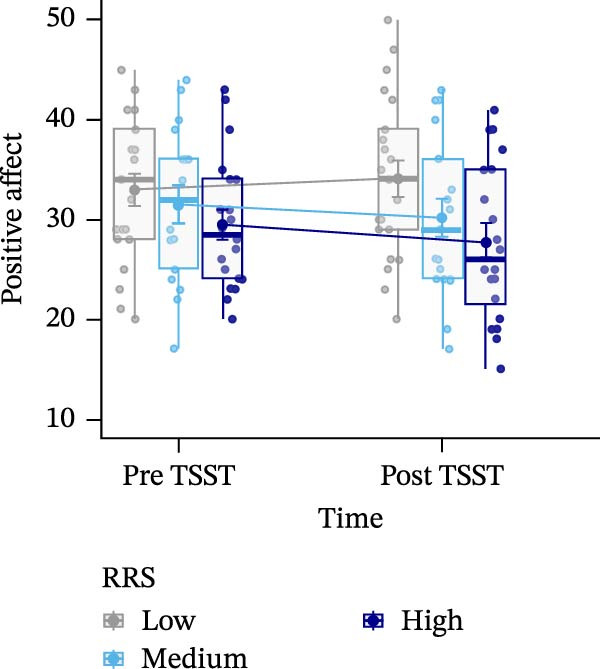
(B)

**Figure:**
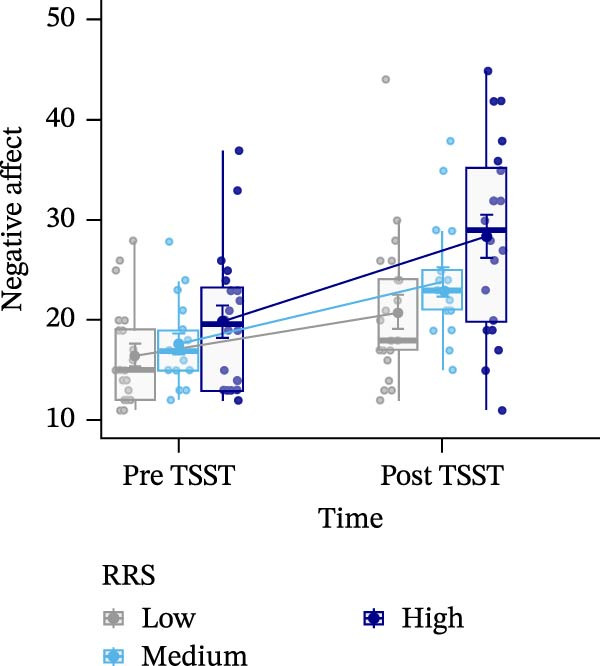
(C)

**Figure:**
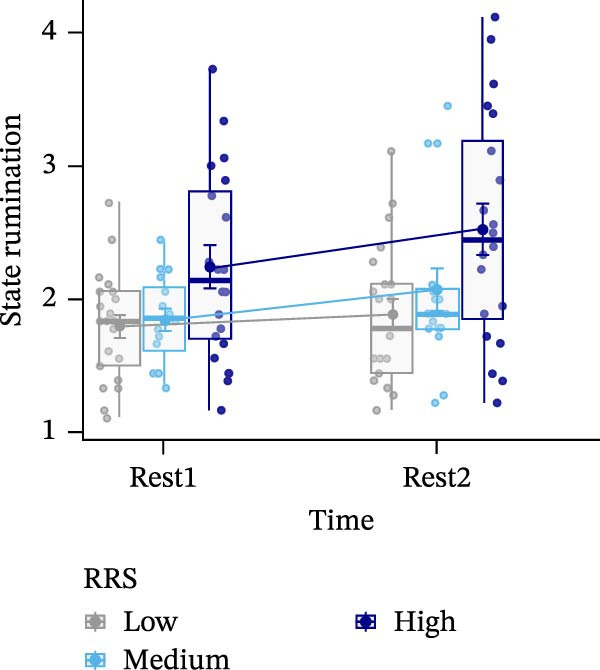
(D)

Figure 4Ratings of the observer‐rated content analysis for the different scales: rehashing bad performance (A), speculation on negative consequences (B), focus on negative affect (C), and reflection (D) dependent on trait rumination group (RRS group). Boxplots illustrate the distribution of the raw data whereby the central line of the boxplots indicates the median and the hinges extend to the 25th and 75th percentiles, respectively. Colored jittered dots represent the individual raw data for each RRS group. The bold dots and connecting line depict the group means.

**Figure:**
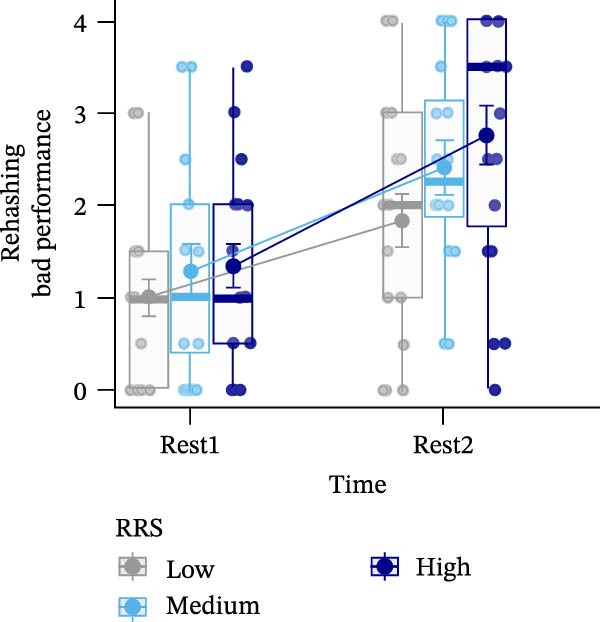
(A)

**Figure:**
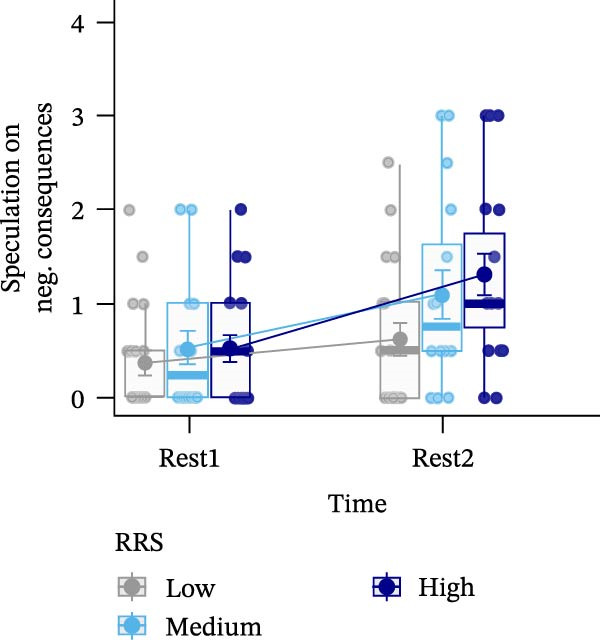
(B)

**Figure:**
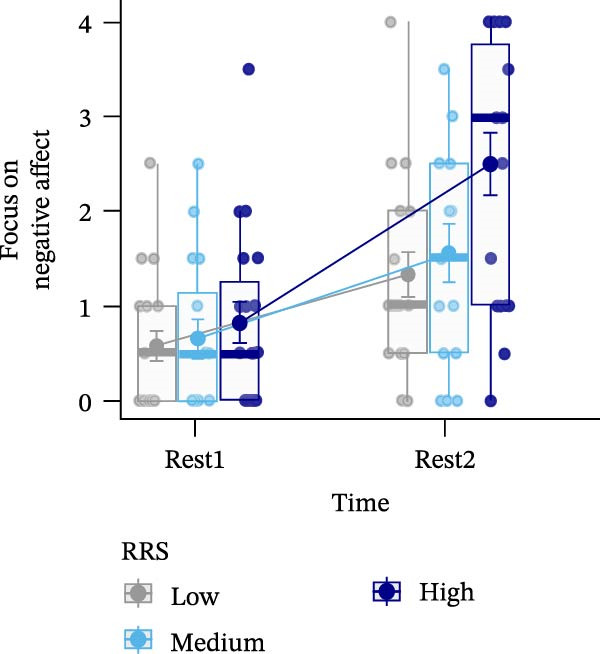
(C)

**Figure:**
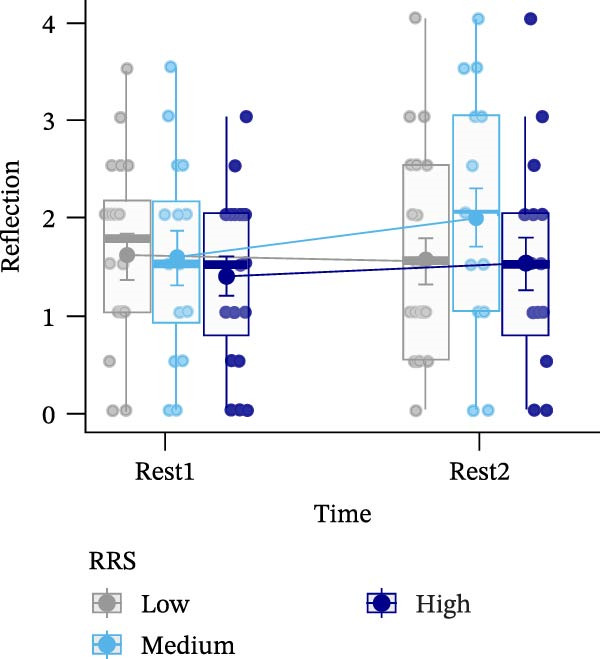
(D)

Multivariate outliers were detected by calculating Mahalanobis distances for each dependent variable (DV: subjective stress as indicated by the VAS ratings, heart rate, self‐censorship, sentiment scores, positive affect, negative affect, state rumination, fNIRS data, observer‐rated rumination) and comparing it to the critical value of the *X* ^2^ distribution (*α* = 0.001). Cases exceeding this threshold were excluded (*n* = 1 for state rumination and *n* = 2 for self‐censorship; *n* = 3 for observer‐rated rumination scale 1).

Repeated measures ANOVAs (rmANOVAs) were applied to each DV, except for the fNIRS data, incorporating an interaction between time and group (low RRS vs. medium RRS vs. high RRS). Participants were grouped into low (mean RRS‐score < 1.82), medium (mean RRS‐score ≥ 1.82 and < 2.36) and high ruminators (mean RRS‐score ≥ 2.36). These cutoffs are based on the combined data of 715 participants including healthy controls and patients with depression from prior studies from our group [38, 39]. To reduce the article’s length, the analysis of math performance and self‐censorship are presented in Supporting Information 1: S10 and S11.

In order to investigate state rumination more thoroughly, we additionally calculated the Reliable Change Index (RCI) by dividing the difference between SRSRQ scores post rest2 and SRSRQ‐scores post rest1 by the standard error of the difference. Then, RCI was compared against a critical difference of 0.55, which was based on a psychometric analysis using a larger sample [15]. To visualize the RCI, we adapted the code of the JTRCI‐package [81]. To reduce the length of the article, this analysis is presented in Supporting Information 1: S12.

For fNIRS data, a rmANOVA was conducted including the factor region of interest (ROI: left IFG, left DLPFC, right IFG, right DLPFC, SAC), time, and RRS group.

Significant findings were followed by post‐hoc analyses corrected for multiple comparisons using Benjamini–Hochberg. Polynomial contrasts (linear and quadratic) were included for interpretative purposes. Analysis of the fNIRS data further included Helmert‐contrasts in case of the factor group. Sphericity violations (Mauchly’s test, *p* < 0.05) were adjusted using Greenhouse‐Geisser estimates if 𝝐 < 0.75, and Huynh–Feldt estimates if 𝝐 > 0.75.

Please note that we also analyzed the data using linear mixed‐effects models with time × group interactions and a random intercept for each participant. The results are largely consistent with the rmANOVAs reported in the manuscript (see Supporting Information 1: S13). The main effects of time and group remain essentially unchanged; however, for heart rate we now additionally observe a time × group interaction. Similarly, for the observer‐rated content analysis (scale 3), we now detect a time × group interaction in addition to the main effects of time and group.

Lastly, we investigated correlations of changes in sentiment scores (rest2–rest1) with changes in cortical oxygenation (arithmetic task of the TSST minus ctl1). We further performed three mediation analyses which aimed to conceptually replicate the analyses of Rosenbaum et al. [49]: We tested whether increases in right IFG‐activation mediated the effect of RRS group on sentiment scores post rest2, negative affect post rest2 or changes in state rumination.

## 3. Results

### 3.1. Sample

The mean age of the sample was 23.47 years (*SD* = 3.87), with 63.8% females. The sample had a BDI‐II of 6.33 (*SD* = 6.35), indicating “no depression” according to the German cutoffs [82], a mean RRS of 2.07 (*SD* = 0.50), and a mean LSAS of 34.06 (*SD* = 20.98).

We categorized the sample into three groups based on their RRS: Low (RRS ≤ 1.82), medium (1.82 < RRS ≤ 2.36), and high RRS (RRS > 2.36). There were no differences among these groups in terms of age or sex ratio (see Table 1); however, they differed significantly in all clinical variables: Symptoms of depression (BDI‐II), trait rumination levels (RRS) and symptoms of social anxiety (LSAS) (see Table 1). More specifically, BDI‐II and RRS scores differed between all three groups (all *p*’s < 0.05). LSAS scores differed between the low and medium as well as low and high RRS group (all *p*’s < 0.05). Medium and high ruminators exhibited increased symptoms of social anxiety [83].

**Table 1:** Demographic variables across the three subsamples.

Variable	Low RRS group (*n* = 21)	Medium RRS group (*n* = 17)	High RRS group (*n* = 20)	Test‐statistic comparing the three groups	Total sample
Age	23.14 (2.52)	23.94 (3.94)	23.40 (4.99)	*F*(2,55) = 0.198, *p* = 0.821, $\eta_{p}^{2}$ = 0.007	23.47 (3.87)
Percent female	61.9%	58.8%	70.0%	*χ^2^* (2) = 0.548, *p* = 0.760	63.8%
BDI‐II	3.00 (3.03)	5.76 (4.85)	10.30 (7.89)	*F*(2,55) = 8.732, *p* < 0.001, $\eta_{p}^{2}$ = 0.241	6.33 (6.35)
RRS	1.53 (0.20)	2.11 (0.11)	2.61 (0.24)	*F*(2,55) = 156.981, *p* < 0.001, $\eta_{p}^{2}$ = 0.851	2.07 (0.50)
LSAS	22.69 (17.66)	36.06 (18.96)	44.30 (20.53)	*F*(2,55) = 6.710, *p* < 0.01, $\eta_{p}^{2}$ = 0.196	34.06 (20.98)

*Note*. Means and standard deviations are provided.

Abbreviations: BDI‐II, Beck’s depression inventory II; LSAS, Liebowitz social anxiety scale; RRS, ruminative response scale.

### 3.2. Stress

We observed a significant main effect of time, *F*(3.922,215.700) = 74.976, *p*  < 0.001, $\eta_{p}^{2}$ = 0.577, with a quadratic polynomial contrast, *F*(1,55) = 35.360, *p* < 0.001, $\eta_{p}^{2}$ = 0.391, indicating stress‐increases due to the TSST, and a main effect of group, *F*(2,55) = 11.767, *p* < 0.001, $\eta_{p}^{2}$ = 0.300. Pairwise comparisons indicated significantly higher (*p* < 0.05) stress in the high RRS group (*M =* 40.74, *SE =* 2.73) compared to the medium RRS group (*M* = 32.06, *SE* = 2.97) and the low RRS group (*M* = 22.23, *SE* = 2.67), and significantly higher stress in the medium compared to the low RRS group (see Figure 2A).

We further investigated the respective consecutive time points using pairwise comparisons and found significant stress increases (*p* < 0.05) between the baseline (*M* = 15.78, *SE* = 1.57) and practicing the TAP (*M* = 29.60, *SE* = 2.32), decreases (*p* < 0.05) between the practice phase and the first resting‐state (*M* = 20.77, *SE* = 1.86), significant increases (*p* < 0.05) again between ctl1 (*M* = 19.17, *SE* = 1.72) and ctl2 (*M* = 35.52, *SE* = 2.62), and between ctl2 and the job interview of the TSST (*M* = 54.55, *SE* = 3.08). Lastly, we observed decreases (*p* < 0.05) between the TSST (*M* = 57.10, *SE* = 3.21) and the second resting‐state (*M* = 20.91, *SE* = 2.08) (see Figure 2A).

### 3.3. Heart Rate

Analyzing heart rates, we found a significant main effect of time, *F*(2.637,110.745) = 58.383, *p*  < 0.001, $\eta_{p}^{2}$ = 0.582, with a quadratic time course, *F*(1,42) = 75.950, *p*  < 0.001, $\eta_{p}^{2}$ = 0.644. Pairwise comparisons of consecutive time points yielded significant increases (*p* < 0.05) between rest1 (*M* = 83.96, *SE* = 1.43) and ctl1 (*M* = 91.60, *SE* = 1.91), ctl1 and ctl2 (*M* = 95.25, *SE* = 2.28), significant decreases (*p* < 0.05) between ctl2 and the anticipation of the TSST (*M* = 87.00, *SE* = 2.28), significant increases (*p* < 0.05) between the anticipation and the TSST job interview (*M* = 102.76, *SE* = 2.83) and significant decreases (*p* < 0.05) after the TSST (*M* = 102.26, *SE* = 2.88) and rest2 (*M* = 83.04, *SE* = 1.48) (Figure 2B).

### 3.4. Sentiment Scores

A significant main effect of group, *F*(2,55) = 4.656, *p* < 0.05, $\eta_{p}^{2}$ = 0.145, was found. Pairwise comparisons yielded significantly (*p* < 0.05) higher sentiment scores in medium (*M* = 0.006, *SE* = 0.002) compared to high ruminators (*M* = 0.000, *SE* = 0.002), *t*(72) = 2.718, *p* < 0.01, *d =* 0.634, and higher sentiment scores in low (*M* = 0.006, *SE* = 0.002) compared to high ruminators, *t*(80) = 2.563, *p* < 0.01, *d* = 0.566 (Figure 3A).

### 3.5. Positive Affect

For positive affect, we observed a significant main effect of RRS group, *F*(2,55) = 3.227, *p*  < 0.05, $\eta_{p}^{2}$ = 0.105. Post‐hoc tests indicated overall significantly (*p* < 0.05) lower positive affect in high ruminators (*M* = 2.60, *SE* = 0.13) compared to low ruminators (*M* = 3.05, *SE* = 0.12) (Figure 3B).

### 3.6. Negative Affect

For negative affect, there was a significant main effect of time, *F*(1,55) = 42.885, *p*  < 0.001, $\eta_{p}^{2}$ = 0.438, indicating stress‐related linear increases in negative affect, and a significant main effect of RRS group, *F*(2,55) = 4.743, *p*  < 0.05, $\eta_{p}^{2}$ = 0.147. Post‐hoc tests indicated overall significantly (*p* < 0.05) higher negative affect in high ruminators (*M* = 2.20, *SE* = 0.12) compared to low ruminators (*M* = 1.69, *SE* = 0.11) (Figure 3C).

### 3.7. State Rumination

We observed a significant main effect of time, *F*(1,54) = 7.798, *p*  < 0.01, $\eta_{p}^{2}$ = 0.126, *d* = 0.76, indicating significant increases in state rumination due to the stress induction, and a main effect of group, *F*(2,54) = 5.412, *p* < 0.01, $\eta_{p}^{2}$ = 0.167, *d* = 0.9. Post‐hoc tests of the main effect of group indicated higher state rumination in the high RRS group (*M* = 2.38, *SE* = 0.12) compared to the medium (*M* = 1.96, *SE* = 0.14) and the low RRS group (*M* = 1.84, *SE* = 0.12) (Figure 3D).

### 3.8. Observer‐Rated Content Analysis

For two scales, “rehashing bad performance” and “speculation on negative consequences”, there was a significant main effect of time (*F*(1,52) = 40.569, *p*  < 0.001, $\eta_{p}^{2}$ = 0.438; *F*(1,52) = 15.777, *p*  < 0.001, $\eta_{p}^{2}$ = 0.233), indicating stress‐related increases for all groups. For “focus on negative affect”, we also observed a significant main effect of time, *F*(1,52) = 45.175, *p* < 0.001, $\eta_{p}^{2}$ = 0.465, and a significant main effect of group, *F*(2,52) = 3.648, *p*  < 0.05, $\eta_{p}^{2}$ = 0.123. Post‐hoc tests indicated significantly (*p* < 0.05) higher negative affect for high (*M* = 1.67, *SE* = 0.19) compared to low ruminators (*M* = 0.96, *SE* = 0.19). Lastly, in case of the “reflection” scale, we observed no significant effects (all *p*’*s* > 0.278) (Figure 4). For a detailed investigation of neural activation dependent on observer‐rated content, we refer to Int‐Veen et al. [84].

### 3.9. Correlation Between Observer‐Rated and Self‐Reported State Rumination

Correlating observer‐rated rumination prior to the stress induction (transcript of rest1) with SRSRQ scores after rest1, we observed significant moderate to strong positive associations for each scale except for scale 4 (reflection). The same was true when we considered change scores (rest2 minus rest1). No significant associations were found for rest2 (see Table 2).

**Table 2:** Correlations of SRSRQ scores with observer‐rated rumination.

Variable	Timepoint	Correlation	*p*‐Value
Scale 1: Rehashing bad performance	rest1	0.456	*p* < 0.001
	rest2	0.076	*p* = 0.291
	rest2–rest1	0.678	*p* < 0.001
Scale 2: Speculation on negative consequences	rest1	0.278	*p* < 0.05
	rest2	0.065	*p* = 0.318
	rest2–rest1	0.522	*p* < 0.001
Scale 3: Focus on negative affect	rest1	0.438	*p* < 0.001
	rest2	0.146	*p* = 0.144
	rest2–rest1	0.60	*p* < 0.001
Scale 4: Reflection	rest1	0.217	*p* = 0.055
	rest2	0.054	*p* = 0.347
	rest2–rest1	−0.199	*p* = 0.073

*Note*. One‐sided Pearson‐correlations. rest2–rest1 = SRSRQ score of rest2 – SRSRQ score of rest1.

### 3.10. Cortical Oxygenation

We observed a significant interaction between time and ROI, *F*(6.265,319.496) = 7.261, *p*  < 0.001, $\eta_{p}^{2}$ = 0.125, and a significant interaction of time and group, *F*(4,102) = 2.864, *p*  < 0.05, $\eta_{p}^{2}$ = 0.101 (Figure 5; see also Supporting Information 1: S14 for brain maps of the contrasts and time series). Post‐hoc tests of the interaction of time and group indicated significant increases in cortical oxygenation between ctl1 and the TSST for low ruminators compared to medium and high ruminators (linear contrast time by group low RRS vs. (medium RRS and high RRS): *F*(1,55) = 9.882, *p* < 0.01, $\eta_{p}^{2}$ = 0.291). Post‐hoc tests of the interaction between time and ROI yielded significant decreases over time in the right IFG (*F*(1,54) = 6.509, *p* < 0.05, $\eta_{p}^{2}$ = 0.108) and significant increases in the left (*F*(1,54) = 21.190, *p* < 0.001, $\eta_{p}^{2}$ = 0.282) and right DLPFC (*F*(1,54) = 19.100, *p* < 0.001, $\eta_{p}^{2}$ = 0.261) and SAC (*F*(1,54) = 14.290, *p* < 0.001, $\eta_{p}^{2}$ = 0.209). Please note that due to potential different path length factors, we conducted post‐hoc tests separately for each ROI.

### 3.11. Association of Sentiment Scores, Observer‐Rated Rumination, and Cortical Oxygenation

Correlating changes in sentiment scores and observer‐rated rumination (rest2 minus rest1) with changes in cortical oxygenation (arithmetic task of the TSST minus ctl1), we observed no significant correlation in any ROI. According to these results, we did not perform the mediation analysis as in Rosenbaum et al. [49].

## 4. Discussion

The present study aimed to explore the psychological and physiological stress responses elicited by the TSST, utilizing the TAP as a novel method for assessing rumination, and investigating its ecological validity. Participants underwent a 10‐min resting‐state before and after the TSST during which they were seated in a quiet room and instructed to allow their minds to wander while verbalizing their thoughts.

Our results revealed significant increases in stress, state rumination, negative affect, heart rate and linear increases in cortical oxygenation in all ROIs except for the IFG, which supports the effectiveness of our stress induction.

Moreover, significant main effects of RRS group were found for all psychological variables. High ruminators showed higher stress, rumination, and negative affect—and lower positive affect—regardless of time, which was also replicated by the observer‐rated analysis of the transcripts. Importantly, these effects remained robust when the data were additionally analyzed using linear mixed‐effects models. This contrasts the findings of Rosenbaum et al. [49], who observed higher stress‐related increases in negative affect and state rumination (and stress in planned contrasts) among high ruminators. While they used the same questionnaires for negative affect and stress, their state rumination items differed slightly, and they only included low and high ruminators in their sample. When we only compared low and high ruminators and excluded medium ruminators, this did not alter our results. Moreover, according to their RRS and BDI‐II, low and high ruminators of our study and Rosenbaum et al. [49] did not seem to differ systematically. Please further note that our findings are limited to non‐clinical populations. It can be expected that, particularly in clinical samples, the psychological stress response would be more pronounced, and neural activation patterns would likely show the characteristic prefrontal hypoactivation commonly observed in such populations. Future studies should also include a clinical sample to be able to draw conclusions about the full rumination spectrum.

Further, we calculated Reliable Change Indices for state rumination ratings to assess individual changes due to stress induction. 65%–80% of participants showed no reliable change, 5%–10% exhibited reliable decreases, and 15%–30% displayed reliable increases in state rumination. These results only partially align with Rosenbaum et al. [49], where 80% of low ruminators showed no reliable change, 20% exhibited increases, and none showed reliable decreases. High ruminators in their study followed a (marginally significantly) different pattern, with 50% showing reliable increases, 45% showing no change, and 5% showing reliable decreases in state rumination following the stress induction (see Supporting Information 1: S15).

Overall, state rumination increases were more pronounced in Rosenbaum et al. [49] (*F*(1,44) = 19.832, *p* < 0.001, $\eta_{p}^{2}$ = 0.311, *d* = 1.34) than in our study (*F*(1,54) = 7.798, *p* < 0.01, $\eta_{p}^{2}$ = 0.126, *d* = 0.76). Most probably, the mere act of verbalizing thoughts during the TAP may have influenced not only participants’ cognitive and emotional responses but also the content of their spoken thoughts. To date, there is no study that investigates how far the verbalization of thoughts changes rumination, although the phenomenon is well‐known in psychotherapy and “affect labeling”. In psychoanalysis or in sensu exposure methods for post‐traumatic stress disorder (e.g., EMDR), patients are encouraged to verbalize all current thoughts. As those methods reduce symptoms of mental disorders, it might be possible that the verbalizing process has some sort of top‐down regulative effect. Moreover, there is a large body of evidence on the effects of “affect labeling”, showing that verbalizing emotional content works as an implicit emotion regulation strategy (e.g., [85–88]). This is further supported by the transcripts in the current study: One participant, for instance, verbalized: “So I think if I were standing now, I would realize, okay, my legs aren’t quite stable yet, but I feel much better now from talking and reflecting on how the conversation went.” Another said: “We can actually just clear our head a little bit, because somehow I do feel like when you talk, you kind of empty your mind a bit—or also when you write.” Exploring top‐down control in thought verbalization during rumination is promising, but these findings suggest the TAP may influence the very process it aims to measure. Future studies should include a control condition, as all participants in our study underwent the same procedure.

Sentiment analysis showed no effect of time or interaction with RRS group, but high ruminators generally exhibited lower sentiment scores (i.e., more negatively valenced thoughts). This aligns with the lack of group‐by‐time effects in state rumination and negative affect. As the first study using the TAP pre‐ and post‐stress, we replicated findings linking negative thought content to rumination [28, 30].

In the analysis of the fNIRS data, we observed significant increases in cortical oxygenation due to the stress induction in the bilateral DLPFC and SAC. We further replicated previous findings by demonstrating prefrontal hypoactivation under stress in medium and high ruminators compared to low ruminators. Interestingly, however, we observed a relative deactivation in the right IFG. One explanation for this might be an inverse U‐shaped relationship between the recruitment of the right IFG and task demands. If task demands exceed a certain point, activation no longer increases but decreases as a form of depletion. Previous studies have stated an important role of the right IFG in a particular form of executive control, namely inhibitory control [89–94]. Inhibitory control might be needed to refocus attention after miscalculations or distractions by emotional non‐reactivity of the TSST panel back to task‐relevant stimuli. Interestingly, rumination has also been related to deficits in cognitive control and inhibition [9], which further supports the idea of the right IFG playing a significant role. This can be easily integrated when functional connections to the limbic system are considered: High ruminators may exhibit heightened limbic activation in response to increased negative affect. This intensified emotional reactivity during the TSST may interfere with the recruitment of prefrontal regions in order to regulate these emotions and redirect attention to task‐relevant stimuli, potentially reflecting a disruption of top‐down cognitive control mechanisms. Unfortunately, using fNIRS, we are limited to the investigation of cortical areas; however, a functional connectivity analysis using for instance fMRI would be interesting to investigate.

Going further, it might be interesting to explore whether findings of neural activation under stress align with neural activation during the resting‐state‐measurements. Although it is out of the scope of this paper, this idea is inspired by a very recent publication by Misaki et al. [95] that found different predictive value of functional connectivity during resting‐state‐measurements versus functional connectivity during a task where negative thinking state was induced. Trait‐rumination in patients was only predicted by negative‐thinking‐state functional connectivity, not from resting‐state functional connectivity. Applied to this study, it might be interesting to investigate on the one hand functional brain activation and on the other hand compare neural patterns during TAP in resting‐state‐measurement before and after the stress induction.

In line with this, an important methodological consideration concerns the timing of rumination assessment. In the present study, ruminative thoughts were not measured during the TSST itself but in real time during the resting‐state period immediately following the stress induction. This approach was chosen because the post‐stress recovery phase is considered a key window in which stress‐induced rumination is likely to occur. Moreover, during the TSST participants are expected to focus primarily on task demands (e.g., speech preparation and performance), which may limit the occurrence or reportability of ruminative thoughts at that moment. Importantly, the TAP captures ongoing thought content as it unfolds, thereby avoiding the limitations of purely retrospective self‐report measures. Future studies could further extend this approach by attempting to assess cognitive processes concurrently with the stress induction (e.g., using think aloud paradigms), although implementing such measures during the TSST may be methodologically challenging and could potentially interfere with the stress manipulation.

## 5. Limitations

When interpreting the results of the sentiment analysis, several limitations should be considered. First, the lexicon employed includes only 1650 positive and 1800 negative words, which means that certain words (e.g., feelings of guilt) are missing and therefore not assigned a sentiment score. The current sentiment analysis is further limited to assessing the valence of verbalized thoughts, capturing only one dimension of ruminative thinking, which is why we additionally conducted an observer‐rated content analysis. Sentiment analysis might further not adequately capture negations unless explicit negation words are used and is unable to detect irony or sarcasm. For a more sophisticated sentiment analysis, advanced methods such as transformer‐based models like bidirectional encoder representations from transformers (BERT) or generative pre‐trained transformer (GPT), and deep neural networks, would offer more reliable results.

This study replicated the well‐documented prefrontal hypoactivation during the TSST in high ruminators [38, 49, 96]. Importantly, we demonstrated that the TAP is a viable method for capturing state rumination, as reflected in its sensitivity to TSST‐induced changes and its convergence with self‐reported measures. These findings offer initial validation of the TAP as a potential behavioral tool for assessing rumination in dynamic contexts. However, the TAP should be used with caution, as the verbalization might influence the ruminative process itself, reflected by reduced response rates to the TSST in the sample at hand. Our findings highlight the importance of including appropriate control conditions in future studies. For example, studies could incorporate a comparison group that does not perform a think aloud procedure, or use a staggered design in which brief rest periods alternate with state rumination assessments and the TAP. Such designs would help to separate potential effects of the measurement procedure itself from the underlying cognitive and emotional processes of interest. To advance the utility of the TAP, future work should focus on developing automated procedures for rumination ratings of the transcripts. In follow‐up studies, we plan to investigate how specific facets of rumination relate to neural activity patterns during acute stress.

Another limitation of the present study is that we did not include cortisol measurements. Cortisol is a well‐established marker of the response to stress [97] and has been widely used to investigate physiological correlates of rumination and stress‐related psychopathology [98]. Including cortisol data could have provided additional insights into the relationship between real‐time ruminative thought processes and physiological stress responses. Future studies with cortisol sampling would allow for a more comprehensive understanding of how cognitive, affective, and endocrine responses interact and may help identify biomarkers relevant for intervention and prevention strategies targeting maladaptive rumination.

Further, participants were categorized into low, medium, and high ruminators based on a trait questionnaire, which does not capture dynamic, time‐dependent changes in rumination. While this limits insights into the temporal unfolding and persistence of ruminative thought processes per se, such questionnaires remain standard practice and have demonstrated high reliability and validity (see Supporting Information 1: S4 for psychometric properties of the Ruminative Response Scale used). Importantly, trait rumination was strongly associated with state measures, such as observer‐rated thought content derived from the TAP, supporting the validity of the TAP transcripts in reflecting actual state rumination processes, given the well‐established link between trait and state rumination. In another manuscript on this data, we further conducted additional analyses of the transcripts to examine the extent to which trait rumination—assessed via the Ruminative Responses Scale (RRS)—is associated with the same neural stress responses as those observed in participants identified as ruminators based on transcript content [84].

A further limitation of our study is the lack of a control group. All participants underwent the TSST between the pre‐ and post‐TAP measurements, which means that observed changes cannot be unequivocally attributed to the stressor itself. Some effects may also reflect unspecific factors such as the passage of time, repeated measurements, fatigue, or habituation. Future studies could include a control or sham‐stressor condition to more precisely isolate the effects of acute social‐evaluative stress.

Another limitation of our study is that we did not include additional covariates such as age, time of day, physical activity, BMI, or sex in the main analyses. While our sample was relatively homogeneous and screened for major confounding factors, we acknowledge that these variables could influence both neural and behavioral stress responses. Future studies should systematically control for these factors to assess their potential moderating effects and to further validate the specificity of the observed associations between stress‐induced neural changes and state rumination.

Our findings have several potential real‐world implications. They demonstrate that analyzing verbalized thoughts provides a more ecologically valid assessment of ruminative processes, allowing researchers to track changes over time across different contexts. At the same time, engaging in the TAP appears to facilitate emotion regulation and may itself influence rumination. This suggests that similar exercises could be applied in therapeutic settings to help patients become more aware of their ruminative processes and develop strategies to manage them.

Further research should further examine the temporal dynamics of emotion regulation during the TAP, to evaluate its potential not only as an assessment tool but also as a possible intervention for state rumination. Integrating advanced linguistic models—capable of identifying nuanced emotional content, including negation and sarcasm—may deepen our understanding of the complex interplay between stress, rumination, and emotion regulation.

## 6. Conclusions

This study demonstrates that the TAP is a promising method for capturing state rumination in real time. Its sensitivity to stress‐induced changes and its convergence with self‐report measures provide initial validation of the TAP as a potentially ecologically valid behavioral assessment tool. However, the act of verbalizing thoughts may itself influence the ruminative process, as suggested by reduced stress responses in some participants. These findings highlight both the potential and the limitations of the TAP in stress research.

## Funding

This research was financially supported by the Deutsches Zentrum für Psychische Gesundheit (DZPG). Open Access funding enabled and organized by Projekt DEAL.

## Conflicts of Interest

The authors declare no conflicts of interest.

## Supporting Information

Additional supporting information can be found online in the Supporting Information section.

## Supporting information


**Supporting Information 1** S1: Inclusion and exclusion criteria of the study. S2: CONSORT‐diagram of the study. S3: Details on a priori and post hoc power analyses. S4: Psychometric properties and details of the used questionnaires. S5: Written instructions of the Think Aloud Paradigm. S6: Details on the Near‐Infrared Spectroscopy measurement. S7: Details on the electrocardiogram measurement. S8: Details on the calculation of sentiment scores. S9: Inter‐rater‐reliability of the qualitative analysis. S10: Results of the rmANOVAs of math performance. S11: Results on self‐censorship. S12: Reliable change in state rumination. S13: Results of the linear mixed models. S14: Brainmaps of the contrasts of low and high ruminators and Time series of cortical oxygenation in each ROI. S15: Reliable change indices in Rosenbaum et al. [38, 49, 50].

**Supporting Information 2** Syntax_SPSS.doc: provides the SPSS syntax used for the analysis.

## Data Availability

Data and analysis scripts are available upon reasonable request from the corresponding author.
