# Supplementary material for: Rumination Out Loud? Linguistic, Neural, and Psychophysiological Correlates of the Think‐Aloud Paradigm
Source: Depress Anxiety. 2026 Apr 23;2026:8855110. doi: 10.1155/da/8855110 (PMC13106887; doi:10.1155/da/8855110)
Supplement: Supplementary file 1 — Supporting Information 1 S1: Inclusion and exclusion criteria of the study. S2: CONSORT‐diagram of the study. S3: Details on a priori and post hoc power analyses. S4: Psychometric properties and details of the used questionnaires. S5: Written instructions of the Think Aloud Paradigm. S6: Details on the Near‐Infrared Spectroscopy measurement. S7: Details on the electrocardiogram measurement. S8: Details on the calculation of sentiment scores. S9: Inter‐rater‐reliability of the qualitative analysis. S10: Results of the rmANOVAs of math performance. S11: Results on self‐censorship. S12: Reliable change in state rumination. S13: Results of the linear mixed models. S14: Brainmaps of the contrasts of low and high ruminators and Time series of cortical oxygenation in each ROI. S15: Reliable change indices in Rosenbaum et al. [38, 49, 50]. [file DA-2026-8855110-s002.doc]

**Supplementary Material**

**Supplementary material S1:** Inclusion and exclusion criteria of the study

**Inclusion criteria:**

- age between 18 and 40 years
- normal vision (or appropriate correction)
- German as native language or very good knowledge of German

**Exclusion criteria:**

- diabetes mellitus
- renal insufficiency
- untreated hypertension
- history of traumatic brain injury
- cardiac arrhythmia
- acute substance abuse
- adrenal insufficiency
- any acute psychiatric or neurological disorder, including any anomalies in the Screening questionnaire of the Structured Clinical Interview (SCID; First et al., 2015)
- in case of women: pregnancy

**Supplementary material S2:** CONSORT-diagram of the study

**
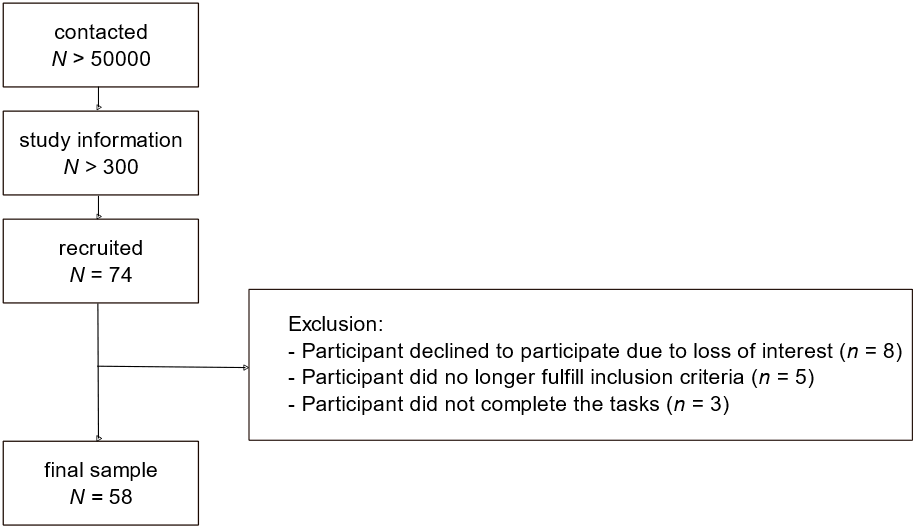
**

**Supplementary material S3:** Details on a priori and post hoc power analyses

**Sensitivity analysis**

A sensitivity analysis was conducted using G*Power 3.1.9.7 for a rmANOVA with three groups (low, medium, and high ruminators) and two measurement points (pre-TSST, post-TSST) (*F*-Tests; ANOVA: repeated measures, between factors). The parameters were: *α* = .05, total sample size = 58, number of groups = 3, number of measurements = 2, correlation of *r* = .6 between repeated measures (based on state rumination scores). The design was sensitive to detect effects of at least *f* = 0.38 (
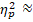
 0.13) with a statistical power of .80. For a target power of .95, the minimum detectable effect size was *f* = 0.47 (
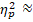
 0.18).

**
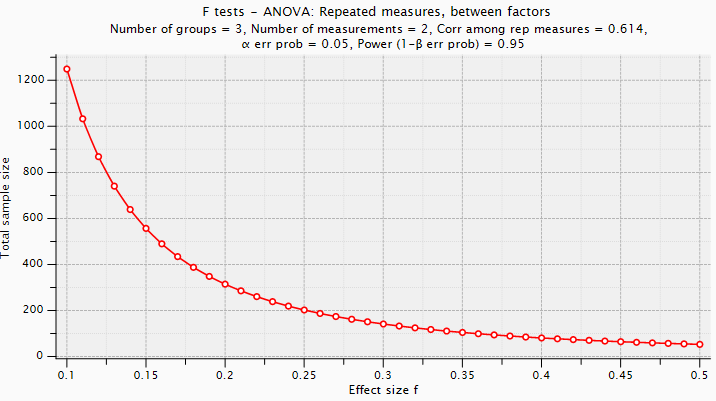
**

**Figure S3:** X–Y plot of sample size in relation to effect size.

**Post-hoc power analysis**

A post-hoc power analysis was conducted using G*Power 3.1.9.7 for a rm
ANOVA with three groups (low, medium, and high ruminators) and two measurement points (pre-TSST, post-TSST) (*F*-Tests; ANOVA: repeated measures, within factors for main effects of time and ANOVA: repeated measures, between factors for main effects of group). The parameters were: *α* = .05, total sample size = 58, number of groups = 3, number of measurements = 2, non-sphericity correction ε = 1. Correlations between repeated measures were derived from the observed data, and effect sizes were extracted from the empirical analyses. The achieved power estimates for all reported effects were ≥ .80, indicating that the study was sufficiently powered despite the modest overall sample size (see table S3).

**Table S3.** Summary of entered parameters and achieved power

| dependent variable | effect | correlation | effect size f  (calculated using 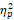) | post-hoc power |
| --- | --- | --- | --- | --- |
| sentiment scores | main effect of group | .791 | 0.412 (.145) | 0.83 |
|  | main effect of time | .791 | n.s. | - |
| self-censorship | main effect of group | .843 | n.s. | - |
|  | main effect of time | .843 | 0.523 (.215) | 1.00 |
| SRSRQ | main effect of group | .614 | 0.448 (.167) | 0.92 |
|  | main effect of time | .614 | 0.380 (.126) | 0.99 |
| PANAS PA | main effect of group | .345 | 0.343 (.105) | 0.80 |
|  | main effect of time | .345 | n.s. | - |
| PANAS NA  main effect of group | main effect of group | .501 | 0.415 (.147) | 0.90 |
|  | main effect of time | .501 | 0.883 (.438) | 1.00 |
| observer-rated thought content: scale 1 | main effect of group | .359 | n.s. | - |
|  | main effect of time | .359 | 0.883 (.438) | 1.00 |
| observer-rated thought content: scale 2 | main effect of group | .199 | n.s. | - |
|  | main effect of time | .199 | 0.551 (.233) | 0.99 |
| observer-rated thought content: scale 3 | main effect of group | .345 | 0.375 (.123) | 0.87 |
|  | main effect of time | .345 | 0.932 (.465) | 1.00 |
| observer-rated thought content: scale 4 | main effect of group | .425 | n.s. | - |
|  | main effect of time | .425 | n.s. | - |

**Note.** Correlations refer to two-sided Pearson-correlations between scores at rest1 and rest2.

**Supplementary material S4:** Psychometric properties and details of the used questionnaires

**Subjective Stress.** Throughout the experiment, participants rated their momentary stress levels eight times using Visual Analogue Scales which were presented on one page so that participants could allow for their last rating: once at baseline, after practicing the TAP, following the first resting-state period, after control task 1, after control task 2, after the job interview component of the TSST, after the arithmetic task of the TSST, and following the second resting-state period. The Visual Analogue Scale ranged from 0 to 100%, with intervals of 10% marked every centimeter. The exact instruction was: Please tick how stressed you currently feel, including the last 5 minutes. And for every stress rating: At the moment, I feel stressed:


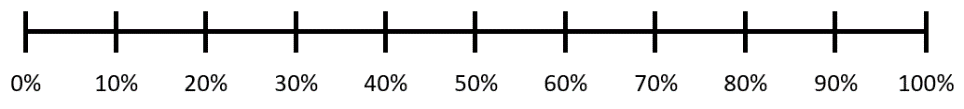


**RRS.** Trait ruminative tendencies were assessed using the Ruminative Response Scale (RRS; Treynor and Gonzalez, 2003). This scale consists of 22 items rated on a 4-point Likert scale, ranging from 1 = "almost never" to 4 = "almost always", yielding a total score between 22 and 88. Please note, that in our analysis, we computed mean RRS-scores, ranging between 1 and 4, respectively. High internal consistency has been consistently reported across various studies and samples (Cronbach’s α = .88; Just and Alloy, 1997; Kasch et al., 2001; Moberly and Watkins, 2008; Nolen-Hoeksema and Morrow, 1991), including studies using the German version of the RRS (Cronbach’s α = .89–.92; Wahl et al., 2011). Test-retest reliability varies depending on the sample and time interval: for non-clinical samples, it typically ranges from *r* = .80 (6 months; Nolen-Hoeksema et al., 1994) to *r* = .67 (1 year; Nolen-Hoeksema et al., 1999). In clinical samples, however, reliability tends to be lower, with values ranging from *r* = .36 (6 months; Kasch et al., 2001) to *r* = .47 (1 year; Just and Alloy, 1997).

**BDI-II.** To assess depression symptom severity, we used the Beck Depression Inventory II (BDI-II), a self-report questionnaire originally developed by Beck et al. (1996, 1961) and later translated into German by Hautzinger and colleagues (2009). The questionnaire evaluates the occurrence of 21 symptoms over the past two weeks, with symptom severity measured as a total score ranging from 0 to 63. Cut-off scores are also provided to help interpret total scores (von Glischinski et al., 2019). Psychometric evaluations across various populations and languages have demonstrated high internal consistency (Cronbach's α around 0.9) and good retest reliability (mean interval of 2 weeks; *r* between 0.7 and 0.9) (Wang and Gorenstein, 2013). Notably, the German version has been shown to effectively differentiate between depressed patients and healthy controls (Kühner et al., 2007) and is considered a reliable screening tool for Major Depressive Disorder (Kumar et al., 2002).

**LSAS.** The Liebowitz Social Anxiety Scale (LSAS; Liebowitz, 1987) is a screening tool for social anxiety disorder, consisting of 24 social situations rated on a 4-point Likert scale for both fear (none, mild, moderate, severe) and avoidance (never, occasionally, often, usually) in reference to the past week. The resulting total score, ranging from 0 to 144, has demonstrated excellent psychometric properties (Cronbach's α = 0.95) (Baker et al., 2002). Additionally, the LSAS has been shown to effectively differentiate between clinical and non-clinical samples (Heimberg et al., 1999; Heimberg and Holaway, 2007; Rytwinski et al., 2009), which is why LSAS scores are used in this study as an indicator of social anxiety levels.

**PANAS.** Current positive and negative affect were assessed pre and post stress using the Positive and Negative Affect Schedule (PANAS; Watson et al., 1988). The scale consists of 20 items rated on a 5-point Likert scale, ranging from 1 ("very slightly") to 5 ("extremely"). Both subscales, positive affect (PA) and negative affect (NA), have demonstrated acceptable internal consistency in both clinical and nonclinical samples, with Cronbach's α ranging from 0.85 to 0.86 for the negative affect subscale (NA) and 0.84 to 0.89 for the positive affect subscale (PA) (Crawford and Henry, 2004; Krohne et al., 2016).

**SRSRQ.** Stress-reactive state rumination was assessed both before and after stress using the Stress-Reactive State Rumination Questionnaire (SRSRQ; Int-Veen et al., n.d.). Items were selected based on the psychometric evaluations conducted by the authors of the original questionnaires, with a focus on the most selective items from the scales of interest. The questionnaire consists of 22 items rated on a 5-point Likert scale, ranging from 1 ("not at all") to 5 ("very often"), resulting in a total score between 22 and 110. For the analysis, we computed mean SRSRQ scores, ranging from 1 to 5. Participants were instructed to rate whether the items accurately reflected their mental state during the last resting-state measurement. The SRSRQ exhibits  high internal consistencies (Cronbach’s α > .95) and high test-retest reliability (*r* > 0.91; mean interval of 1-2 months).

**CTQ.** The Childhood Trauma Questionnaire (CTQ; Bernstein et al., 2003) was used to assess self-reported adverse childhood experiences occurring between the ages of 0 and 17 years. This 28-item short version of the original 70-item CTQ (David P. Bernstein et al., 1994) includes 25 clinical items and three validity items designed to screen for denial (e.g., "I had the best family in the world"). The clinical items are organized into five empirically derived subscales: emotional abuse, physical abuse, sexual abuse, emotional neglect, and physical neglect, which are combined to create a total score (range: 25–125). Responses are rated on a 5-point Likert scale ranging from 1 = "never true" to 5 = "very often true". The short version of the CTQ has demonstrated satisfactory psychometric properties. In clinical and non-clinical samples, Bernstein et al. (2003) found a consistent five-factor structure and strong criterion-related validity, as assessed through therapist ratings of maltreatment, patient-provided information, and child protective investigation data. In a community sample, acceptable internal consistency was observed for the total score (Cronbach's α = .91) and subscale scores (ranging from Cronbach’s α = .58 for physical neglect to Cronbach’s α = .94 for sexual abuse; Scher et al., 2001). The original CTQ version has shown high test-retest reliability, with values ranging from *r* = .79 to .81 (David P Bernstein et al., 1994). These findings have been consistently replicated in other studies (Burns et al., 2012, 2010; Huh et al., 2017), and the German translation has shown similar reliability and validity in both clinical and non-clinical samples (Bader et al., 2009; Klinitzke et al., 2012; Wingenfeld et al., 2010).

**Supplementary material S5: Written instructions of the Think Aloud Paradigm**

| **German** | **English** |
| --- | --- |
| Liebe Versuchsperson,  Jetzt folgt eine 10-minütige Ruhemessung, während der Sie einfach nur ruhig sitzen, Ihren Gedanken freien Lauf lassen sollen und alle Gedanken, die Ihnen spontan aufkommen, aussprechen sollen. Um das Gesprochene aufzunehmen, wird eine Audioaufnahme gemacht. Sprechen Sie daher bitte laut und deutlich in das Mikrofon, das vor Ihnen steht. Es ist wichtig, dass Sie versuchen alle Gedanken auszusprechen, also kontinuierlich zu sprechen und keine längeren Pausen zu machen. Bitte lassen Sie zudem während der Ruhemessung die Augen geöffnet.  Während der Ruhemessung wird der*die Versuchsleiter*in den Raum verlassen und erst nach 10 min wieder hereinkommen, sodass Sie ungestört sind.  Sollten Sie Fragen zur Aufgabe haben, wenden Sie sich bitte jetzt an der*dir Versuchsleiter*in. | Dear test subject,  Now follows a 10-minute rest measurement, during which you should simply sit quietly, let your thoughts run free and speak out any thoughts that spontaneously occur to you. An audio recording will be made of what you say. Therefore, please speak loudly and clearly into the microphone in front of you. It is important that you try to express all your thoughts, i.e. speak continuously and do not take any long pauses. Please also keep your eyes open during the resting measurement.  During the resting measurement, the experimenter will leave the room and only come back in after 10 minutes so that you are undisturbed.  If you have any questions about the task, please contact the experimenter now. |

**Supplementary material S6:** Details on the Near-Infrared Spectroscopy measurement

We assessed cortical oxygenation by employing an ETG-4000 Optical Topography System. This system operates at a sampling rate of 10Hz and uses a 46-channel continuous wave multichannel fNIRS system by Hitachi Medical Co., Japan. The probes consisted of two frontal and one parietal probeset, each with a fixed inter-optode distance of 3 cm. These probesets included 28 light emitters (semiconductor lasers) and 15 light detectors (avalanche photodiodes) emitting and detecting light at two distinct wavelengths (695 ± 20 and 830 ± 20 nm) with a power output of 2.0 ± 0.4mW for each wavelength at each optode. The placement of these probesets adhered to the 10-20 reference points Fpz and Cz. Relative changes in oxygenated (O_2_Hb) and deoxygenated (HHb) hemoglobin levels were computed using custom MATLAB2024a scripts that applied the modified Beer-Lambert Law (Sassaroli and Fantini, 2004). Preprocessing of the data included the following steps: Interpolation of single noisy channels, correction of motion artifacts using Temporal Derivative Distribution Repair (Fishburn et al., 2019), application of Correlation-based signal improvement (Cui et al., 2010), bandpass filtering to eliminate low-frequency baseline drifts (< 0.01 Hz) and high-frequency noise (> 0.1 Hz). To further address artifacts from data correction, another channel interpolation was performed, and performed a Principal Component Analysis across all participants and deleted the first principal component in order to reduce global artifacts. Next, data was z-standardized for facilitated between-subjects comparability. For data analysis, Event-Related Averages were calculated for each trial, incorporating a 10-second baseline correction and linear detrending. The placement of the probesets and the respective Regions of Interest (ROI) is illustrated in figure S6 and table S6.

Finally, we exported the data for each of our ROIs: the left and right IFG, left and right DLPFC, and SAC. Specifically, event-related averages were computed for each 40 s trial of ctl1, ctl2 and the arithmetic task of the TSST (window range 0-65 s; peak window 5-40 s; average window 0-48 s) including a 10 s baseline correction and linear detrending.

Scalp-brain correspondence was estimated based on the research of Okamoto et al. (2004), Okamoto and Dan (2005), and Singh et al. (2005).


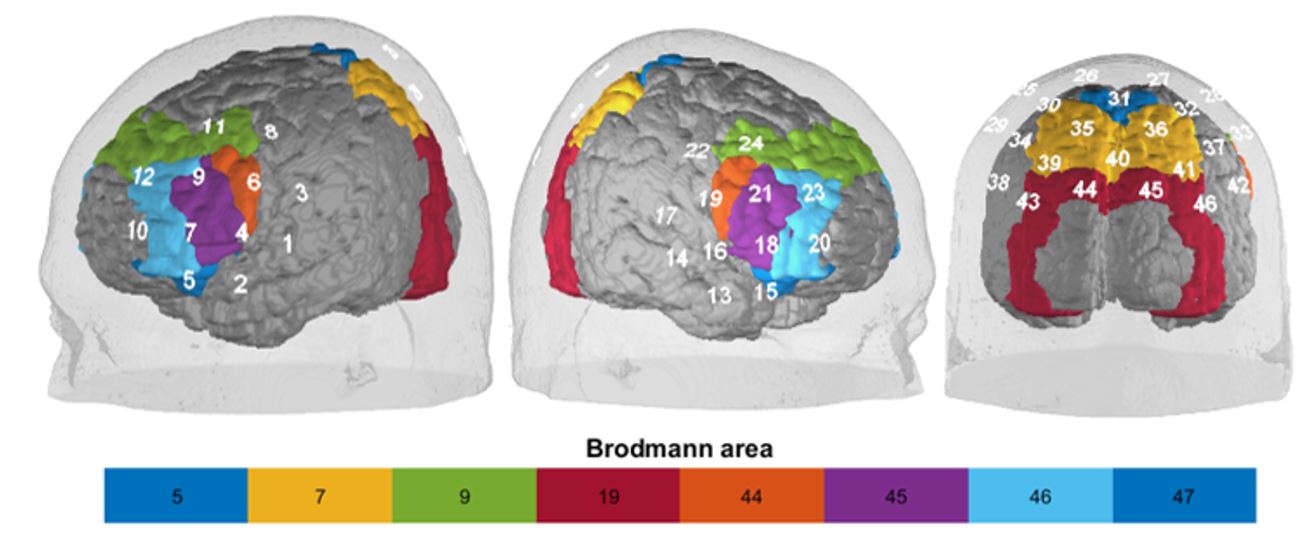


**Figure S6.** Placement of the probesets. Note that the corresponding numbers represent the channels (located midway between emitter and detector).

**Table S6:** Definition of Regions of Interest

| ROI | channel |
| --- | --- |
| left IFG | 7 9 6 |
| right IFG | 18 21 19 |
| left DLPFC | 10 12 11 |
| right DLPFC | 20 23 24 |
| SAC | 27 26 25 28 30 31 32 35 36 |

**Note.** IFG = Inferior Frontal Gyrus, DPFC = Dorsolateral Prefrontal Cortex, SAC = Somatosensory Association Cortex.

**Supplementary material S7:** Details on the electrocardiogram measurement

Throughout the experimental session, heart rate was monitored using three 8mm-diameter Ag/AgCl-ring-electrodes. These electrodes were attached to the participants' skin above the right collarbone, below the left costal arch, and as a reference point below the neck. ECG was recorded with a BrainAmp-EXG-amplifier and BrainVisionRecorder software from Brain Products in Munich, Germany, operating at a sampling rate of 1000Hz. The acquired data underwent preprocessing and analysis using Brain Vision Analyzer 2.1 and MATLAB 2024a. The preprocessing steps encompassed band-pass filtering within the range of 1-30Hz with a slope of 48dB/octave and a time constant of 0.1591549 s. Additionally, a notch filter at 50Hz was applied to eliminate power line artifacts. Finally, the average time interval between consecutive R-peaks was calculated for each recorded condition, expressed in beats per minute.

**Supplementary material S8:** Details on the calculation of sentiment scores

Using R-Version 4.3.1, we conducted a sentiment-analysis as follows: Initially, the raw text data from each participant in each condition (rest1 and rest2) were automatically extracted and split into sentences. For each sentence, every word from a predefined German lexicon (SentiWS version 2.0 including 1650 positive and 1800 negative words provided by the University of Leipzig; Remus et al., 2010) was searched. If a token was found in the sentence, its sentiment-score was added to a running total. If a negation word was found within a clause, the score of that clause was inverted (multiplied by -1). Clauses separated by conjunctions were analyzed individually, ensuring that negations affected only the sentiment-score of the corresponding clause. Lastly, the sentiment-score was divided by the number of token occurrences. This normalization ensures that the score reflects the average sentiment per token rather than just the total.

**Supplementary material S9:** Inter-Rater-Reliability of the qualitative analysis

| Variable | Cohen’s 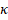 unweighted | Cohen’s 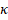 weighted |
| --- | --- | --- |
| Scale 1 raw data: rehashing bad performance | 0.402 | 0.708 |
| Scale 2 raw data: speculating on negative consequences | 0.292 | 0.439 |
| Scale 3 raw data: focus on negative affect | 0.299 | 0.697 |
| Scale 4 raw data: reflection | 0.273 | 0.375 |
| Scale 1 change score: rehashing bad performance | 0.258 | 0.687 |
| Scale 2 change score: speculating on negative consequences | 0.164 | 0.465 |
| Scale 3 change score: focus on negative affect | 0.166 | 0.678 |
| Scale 4 change score: reflection | 0.272 | 0.266 |

**Supplementary material S10:** Results of the rmANOVAs of math performance

**Number of calculations.**

Checking for multivariate outliers using Mahalanobis distances (*p* < .001), no participant had to be excluded. We observed a significant main effect of time, *F*(1.360, 74.800) = 566.352, *p* < .001,
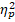
 = .911, but no significant main effect of group, *F*(2, 55) = 0.524, *p* = .595,
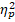
 = .019, nor a significant interaction of time and group, *F*(2.720, 74.800) = 2.108, *p* = .109,
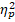
 = .071. Polynomial contrasts indicated a quadratic time course, *F*(1, 55) = 1245.155, *p* < .001,
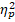
 = .958. Benjamini-Hochberg-corrected pairwise comparisons indicated significant decreases (*p* < .05) in the number of performed calculations between control task 1 (*M* = 23.19, *SE* = 0.19) and control task 2 (*M* = 10.49, *SE* = 0.37) and significant increases between control task 2 and the arithmetic task of the TSST (*M* = 13.55, *SE* = 0.46) (see figure S10A).

**Number of errors.**

Checking for multivariate outliers using Mahalanobis distances (*p* < .001), no participant had to be excluded. We observed a significant main effect of time, *F*(1.734, 95.371) = 190.480, *p* < .001,
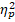
 = .776, but no significant main effect of group, *F*(2, 55) = 0.557, *p* = .576,
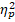
 = .020, nor a significant interaction of time and group, *F*(3.468, 95.371) = 0.699, *p* = .575,
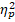
 = .025. Polynomial contrasts indicated a quadratic time course, *F*(1, 55) = 41.155, *p* < .001,
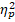
 = .428. Benjamini-Hochberg-corrected pairwise comparisons indicated significant increases (*p* < .05) in the number of performed calculations between control task 1 (*M* = 1.21, *SE* = 0.19) and control task 2 (*M* = 3.41, *SE* = 0.33) as well as between control task 2 and the arithmetic task of the TSST (*M* = 9.95, *SE* = 0.52) (see figure S10B).

**
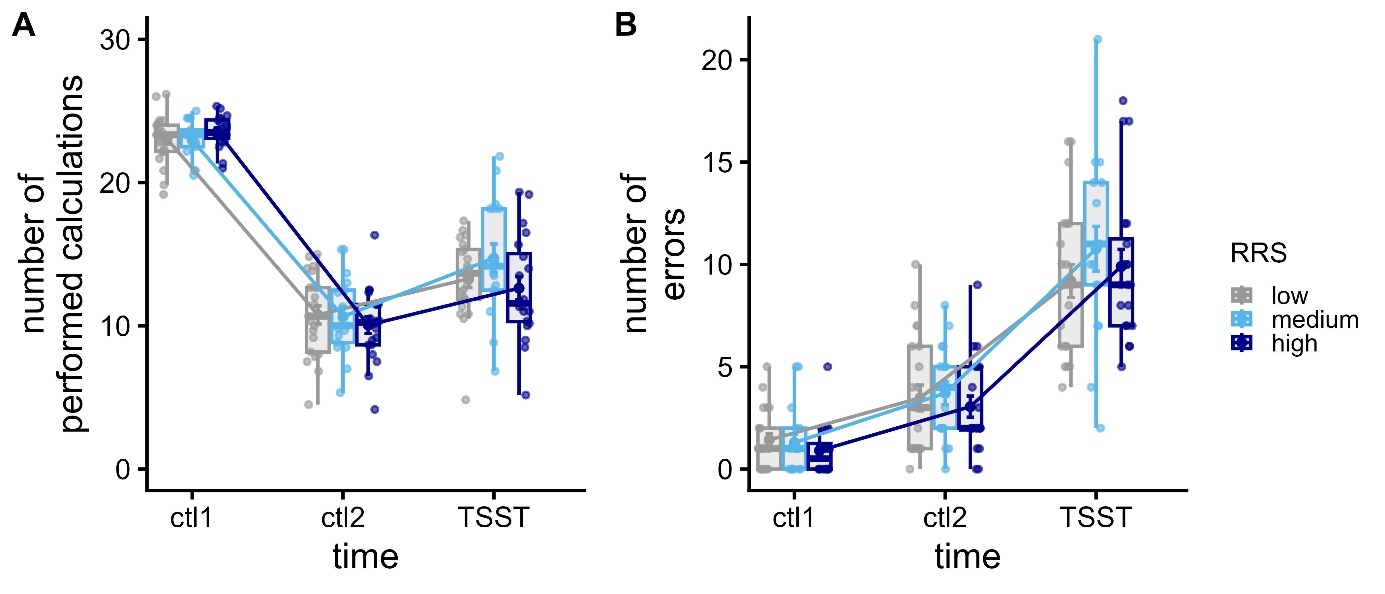
**

**Figure S10.** Line plot of the number of performed calculations (A) and the number of errors (B) dependent on trait rumination group (RRS group). Boxplots illustrate the distribution of the raw data whereby the central line of the boxplots indicates the median and the hinges extend to the 25th and 75th percentiles, respectively. Colored jittered dots represent the individual raw data for each RRS group. The bold dots and connecting line depict the group means.

**Supplementary material S11:** Results on self-censorship

There was a significant main effect of time, *F*(1,53) = 14.499, *p < .*001,
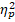
 = .215, indicating overall decreases in how far participants censored themselves from rest1 (*M* = 20.25%, *SD* = 16.82%) to rest2 (*M* = 15.50%, *SD* = 17.24%) (figure S11). On average, participants spoke 1370 words (*SD* = 432) during rest1, and 1359 (*SD* = 387) during rest2. The number of words did not significantly differ between rest1 and rest2, *t*(112.64) = 0.149, *p* = .882, *d* = 0.028. These findings were in line with Raffaeli et al. (2021) also using a 10-min resting-state.

**
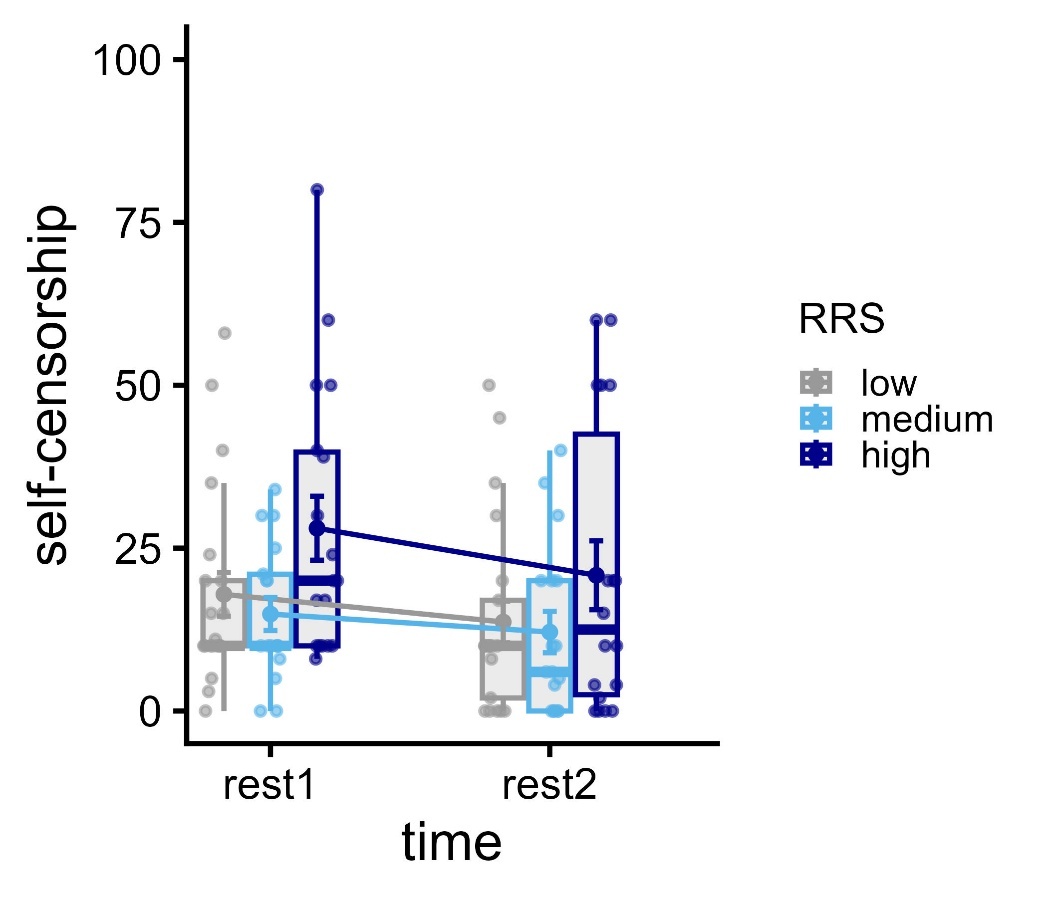
**

**Figure S11.** Self-censorship ratings (0–100%) dependent on trait rumination group (RRS group). Boxplots illustrate the distribution of the raw data whereby the central line of the boxplots indicates the median and the hinges extend to the 25th and 75th percentiles, respectively. Colored jittered dots represent the individual raw data for each RRS group. The bold dots and connecting line depict the group means.

**Supplementary material S12:** Reliable change in state rumination

We computed RCI in order to assess the relative number of participants exhibiting reliable increases, decreases, and no reliable change in SRSRQ scores in each subsample, respectively. The distribution of the three categories was not significantly different between the three subsamples,
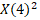
= 1.984, *p* = 0.739: 5-10% showed reliable decreases, 15-30% increases and 65-80% exhibited no reliable changes (see table S12, figure S12).

**Table S12.** Absolute and relative frequencies of reliable change according to Reliable Change Indices (RCI).

|  | reliable decrease | no reliable change | reliable increase | test-statistic |
| --- | --- | --- | --- | --- |
| low RRS  (*n* = 21) | 1  (4.76%) | 17  (80.95%) | 3  (14.29%) | 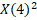 = 1.984, *p* = 0.739 |
| medium RRS  (*n* = 17) | 1  (5.88%) | 11  (64.71%) | 5  (29.41%) |  |
| high RRS  (*n* = 20) | 2  (10.00%) | 13  (65.00%) | 5  (25.00%) |  |

**Note.** Percentages refer to the relative frequencies in the corresponding subsample (low, medium or high ruminators) and the test-statistic indicated
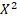
-test comparing the distribution of RCI categories in the subsamples.

**
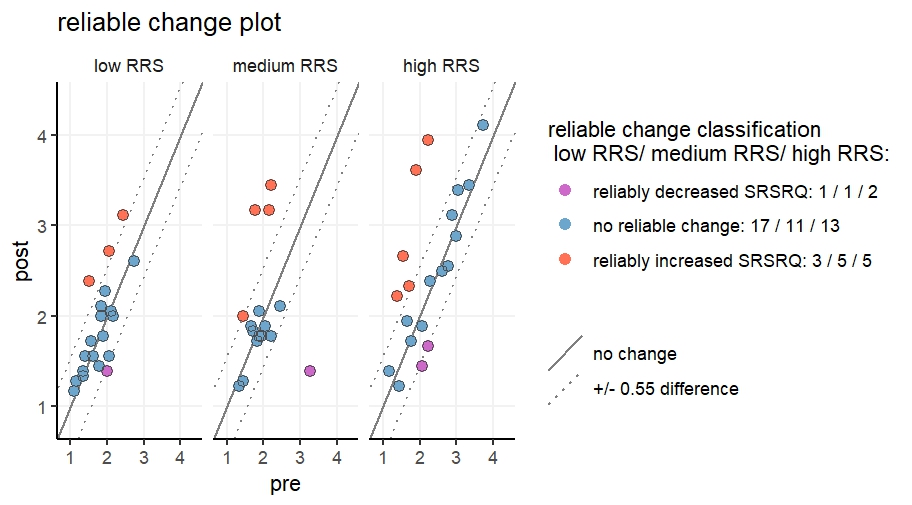
**

**Figure S12.** Reliable change index plotted for changes in state rumination previous to the stress-induction (rest1) to after the stress-induction (rest2) dependent on RRS group.

**Supplementary material S13:** Results of the linear mixed models

To investigate the robustness of the results, we additionally fitted linear mixed models with time × group interactions and a random intercept for each participant.

**Stress**

| effects in the rmANOVA | effects in the lmer |
| --- | --- |
| - main effect of time, *F*(3.922,215.700) = 74.976, *p < .*001, 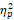 = .577 - main effect of group, *F*(2,55) = 11.767, *p* < .001, 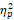 = .300 | - main effect of time, 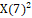 = 518.595, *p* < .001 - main effect of group, 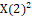 = 23.533, *p* < .001 |

**Heart rate**

| effects in the rmANOVA | effects in the lmer |
| --- | --- |
| - main effect of time, *F*(2.637,110.745) = 58.383, *p < .*001, 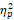 = .582 | - interaction of time and group, 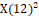 = 23.084, *p* < .05 |

**Sentiment scores**

| effects in the rmANOVA | effects in the lmer |
| --- | --- |
| - main effect of group, *F*(2,55) = 4.656, *p* < .05, 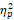 = .145 | - main effect of group, 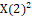 = 9.014, *p* < .05 |

**Positive affect**

| effects in the rmANOVA | effects in the lmer |
| --- | --- |
| - main effect of group, *F*(2,55) = 3.227, *p < .*05, 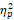 = .105 | - main effect of group, 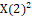 = 6.454, *p* < .05 |

**Negative affect**

| effects in the rmANOVA | effects in the lmer |
| --- | --- |
| - significant main effect of time, *F*(1,55) = 42.885, *p < .*001, 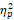 = .438 - main effect of group, *F*(2,55) = 4.743, *p < .*05, 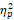 = .147 | - main effect of time, 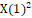 = 42.887, *p* < .001 - main effect of group, 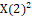 = 9.486, *p* < .01 |

**State rumination**

| effects in the rmANOVA | effects in the lmer |
| --- | --- |
| - main effect of time, *F*(1,54) = 7.798, *p < .*01, 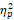 = .126, *d* = 0.76 - main effect of group, *F*(2,54) = 5.412, *p* < .01, 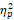 = .167, *d* = 0.9 | - main effect of time, 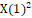 = 7.608, *p* < .01 - main effect of group, 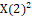 = 10.824, *p* < .01 |

**Observer-rated content analysis: rehashing bad performance**

| effects in the rmANOVA | effects in the lmer |
| --- | --- |
| - main effect of time, *F*(1,52) = 40.569, *p < .*001, 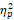 = .438 | - main effect of time, 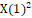 = 38.933, *p* < .001 |

**Observer-rated content analysis: speculation on negative consequences**

| effects in the rmANOVA | effects in the lmer |
| --- | --- |
| - main effect of time, *F*(1,52) = 15.777, *p < .*001, 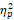 = .233 | - main effect of time, 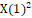 = 15.066, *p* < .001 |

**Observer-rated content analysis: focus on negative affect**

| effects in the rmANOVA | effects in the lmer |
| --- | --- |
| - main effect of time, *F*(1,52) = 45.175, *p < .*001, 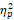 = .465 - main effect of group, *F*(2,52) = 3.648, *p < .*05, 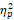 = .123 | - interaction of time and group, 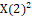 = 6.207, *p* < .05 |

**Observer-rated content analysis: reflection**

| effects in the rmANOVA | effects in the lmer |
| --- | --- |
| - n.s. | - n.s. |

**Cortical oxygenation**

| effects in the rmMANOVA | effects in the lmer |
| --- | --- |
| - interaction between time and ROI, *F*(6.265,319.496) = 7.261, *p < .*001, 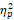 = .125 - interaction of time and group, *F*(4,102) = 2.864, *p < .*05, 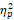 = .101 | - interaction between time and ROI, 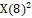 = 31.881, *p* < .001 - interaction between time and group, 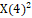 = 14.116, *p* < .01 |

**Self-censorship**

| effects in the rmANOVA | effects in the lmer |
| --- | --- |
| - main effect of time, *F*(1,53) = 14.499, *p < .*001, 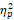 = .215 | - main effect of time, 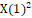 = 14.666, *p* < .001 |

**Performance: Number of calculations**

| effects in the rmANOVA | effects in the lmer |
| --- | --- |
| - main effect of time, *F*(1.360, 74.800) = 566.352, *p* < .001, 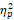 = .911 | - main effect of time, 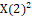 = 1147.888, *p* < .001 |

**Performance: Number of errors**

| effects in the rmANOVA | effects in the lmer |
| --- | --- |
| - main effect of time, *F*(1.734, 95.371) = 190.480, *p* < .001, 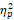 = .776 | - main effect of time, 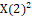 = 379.297, *p* < .001 |

**Supplementary material S14:** Brainmaps of the contrasts of low and high ruminators and Time series of cortical oxygenation in each ROI


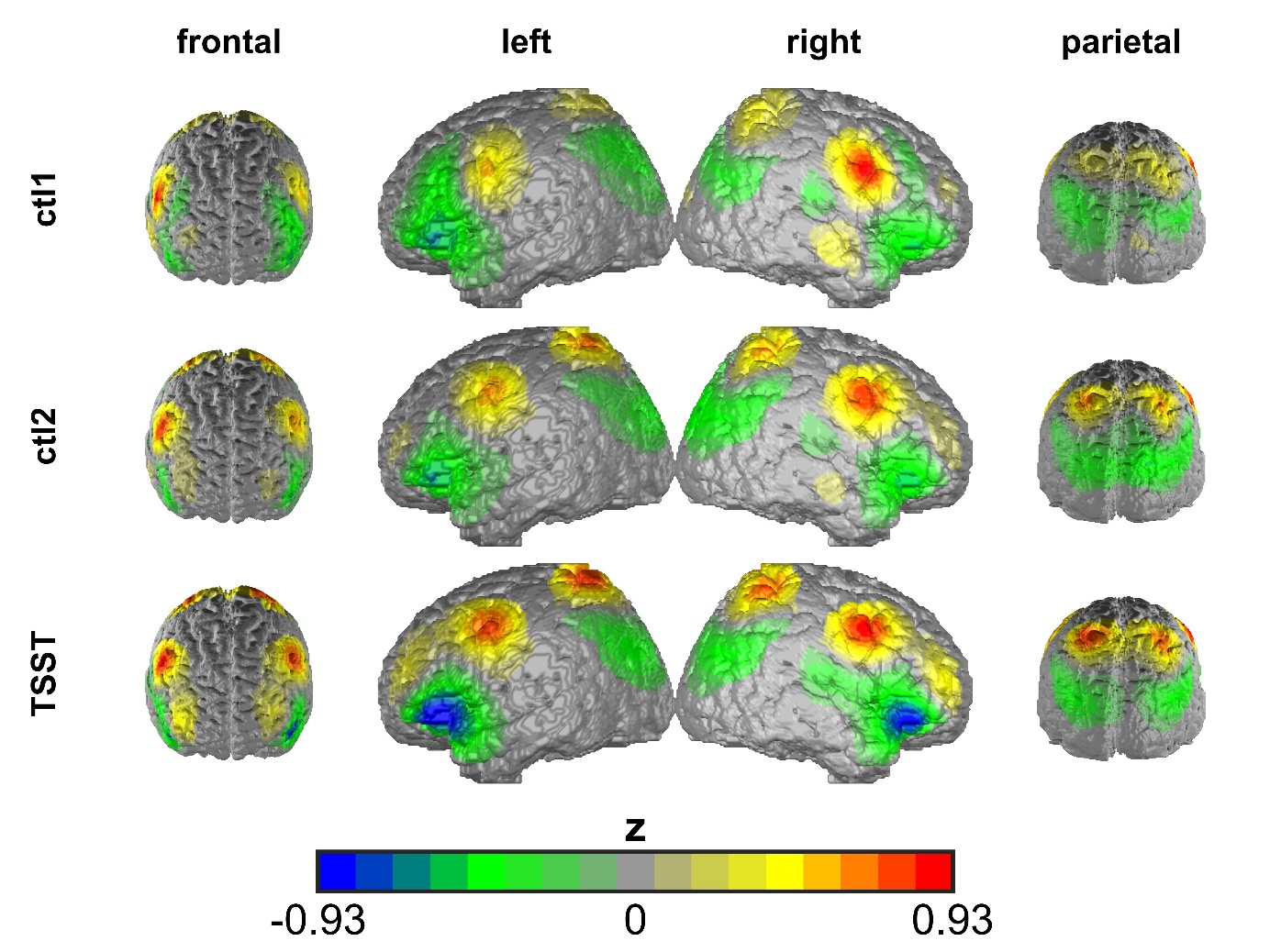


**Figure S14.** Standardized mean cortical oxygenation during control task 1 (ctl1; reading numbers), control task 2 (ctl2; performing calculations without social stress) and during the arithmetic task of the Trier Social Stress Test (TSST; performing calculations under social stress). Warm colors indicate higher cortical oxygenation, cool colors indicate lower cortical oxygenation.

**Illustration of the z-standardized hemodynamic responses** during control task1 (ctl1), control task 2 (ctl2) and the arithmetic task of the TSST (TSST) in the Regions of Interest (ROI): left and right Inferior Frontal Gyrus (IFG), left and right Dorsolateral Prefrontal Cortex (DLPFC) and Somatosensory Association Cortex (SAC). The light shading marks the 40 s trial and the dark shading the 20 s pause to allow the hemodynamic response to recover. Shadings around the hemodynamic curves reflect standard errors of the mean. The baseline includes the 5 s before each trial; 0 s on the x-axis marks the beginning of the trial.


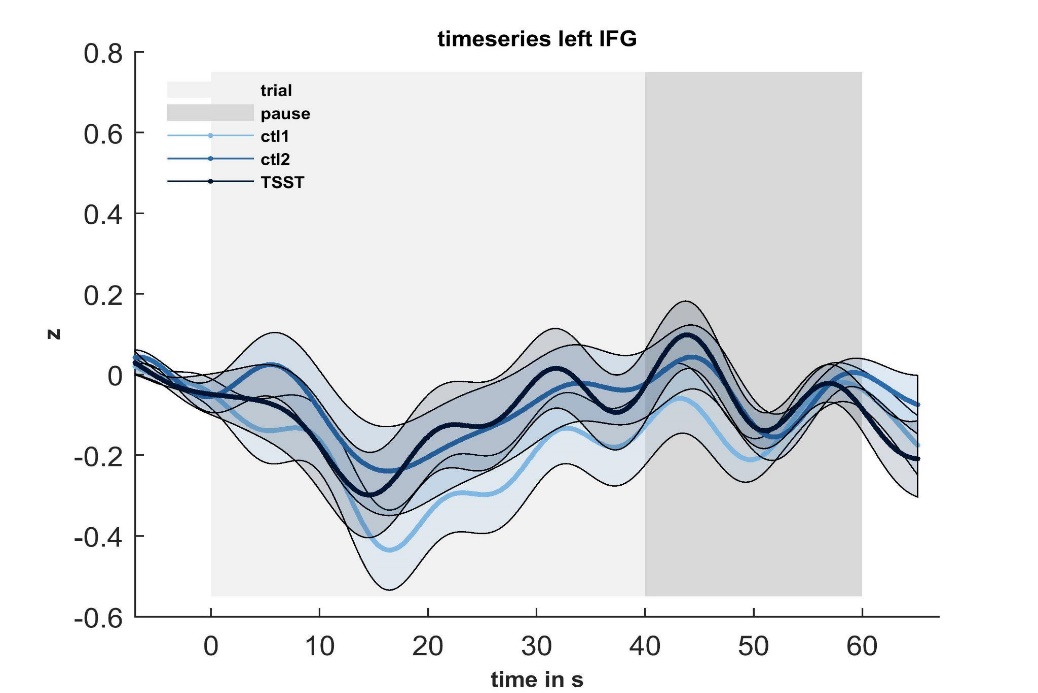

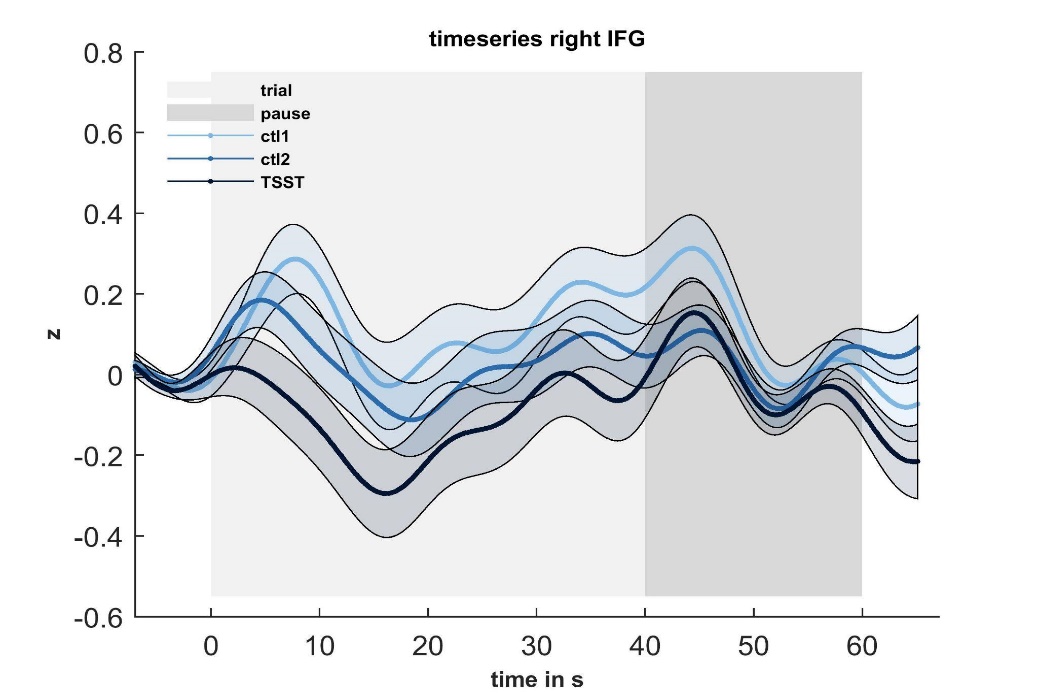

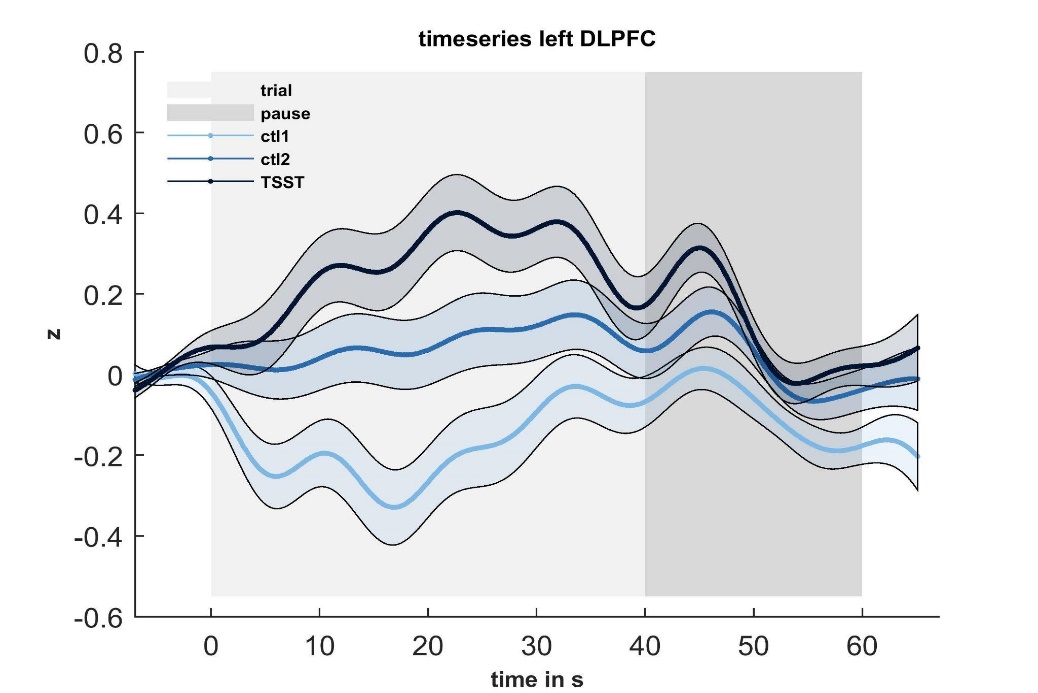

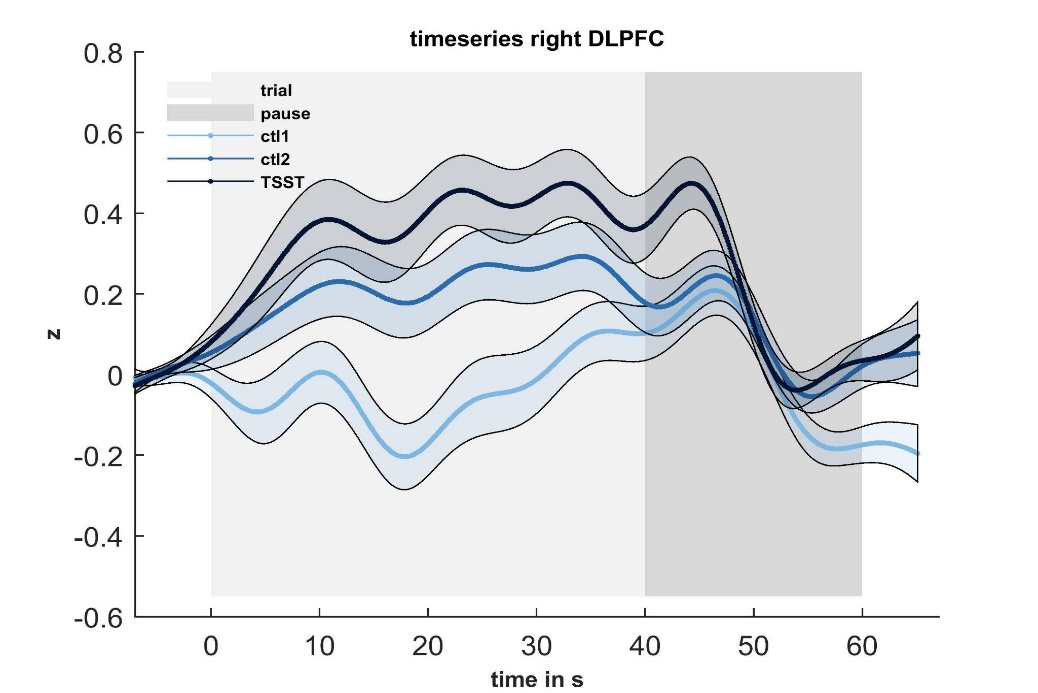

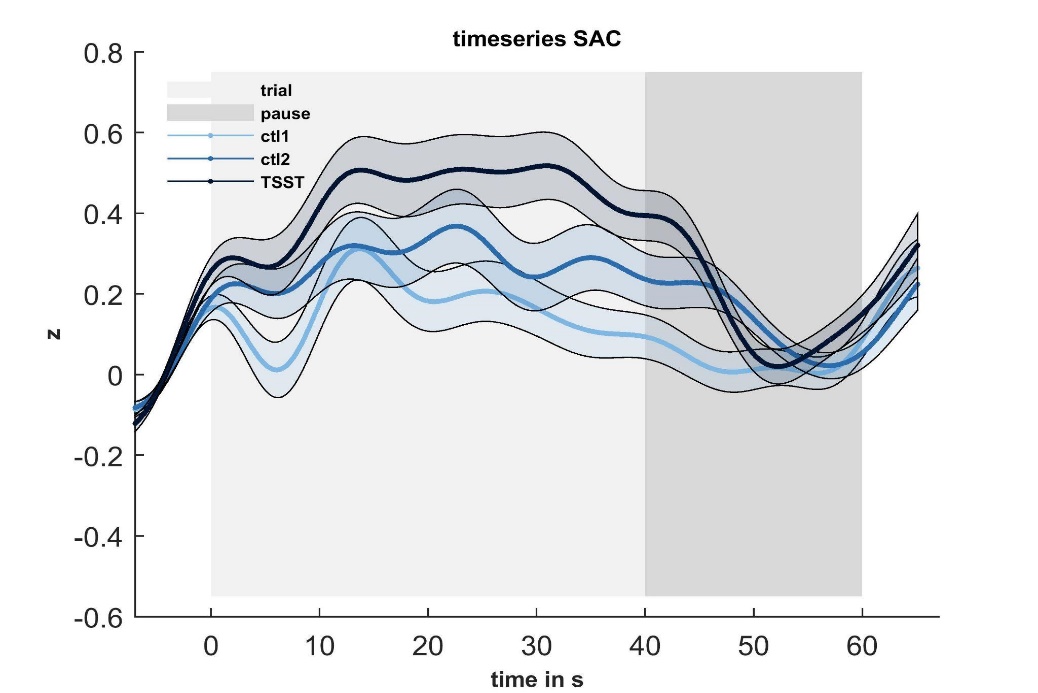


**Illustration of the z-standardized hemodynamic responses** during the arithmetic task of the TSST for low, medium and high ruminators in the Regions of Interest (ROI): left and right Inferior Frontal Gyrus (IFG), left and right Dorsolateral Prefrontal Cortex (DLPFC) and Somatosensory Association Cortex (SAC). The light shading marks the 40 s trial and the dark shading the 20 s pause to allow the hemodynamic response to recover. Shadings around the hemodynamic curves reflect standard errors of the mean. The baseline includes the 5 s before each trial; 0 s on the x-axis marks the beginning of the trial.


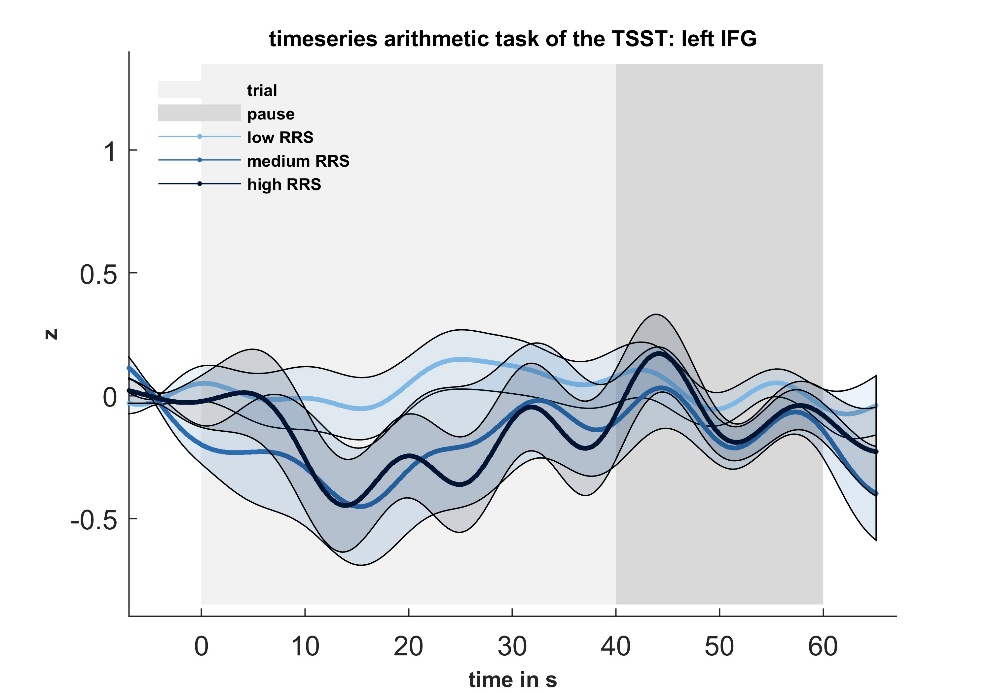

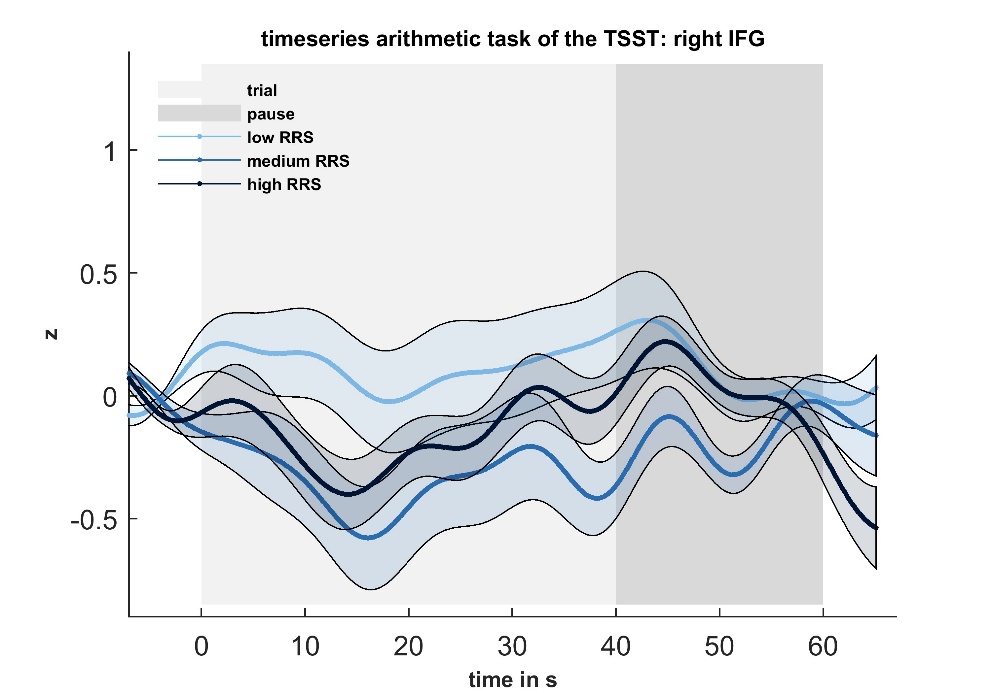

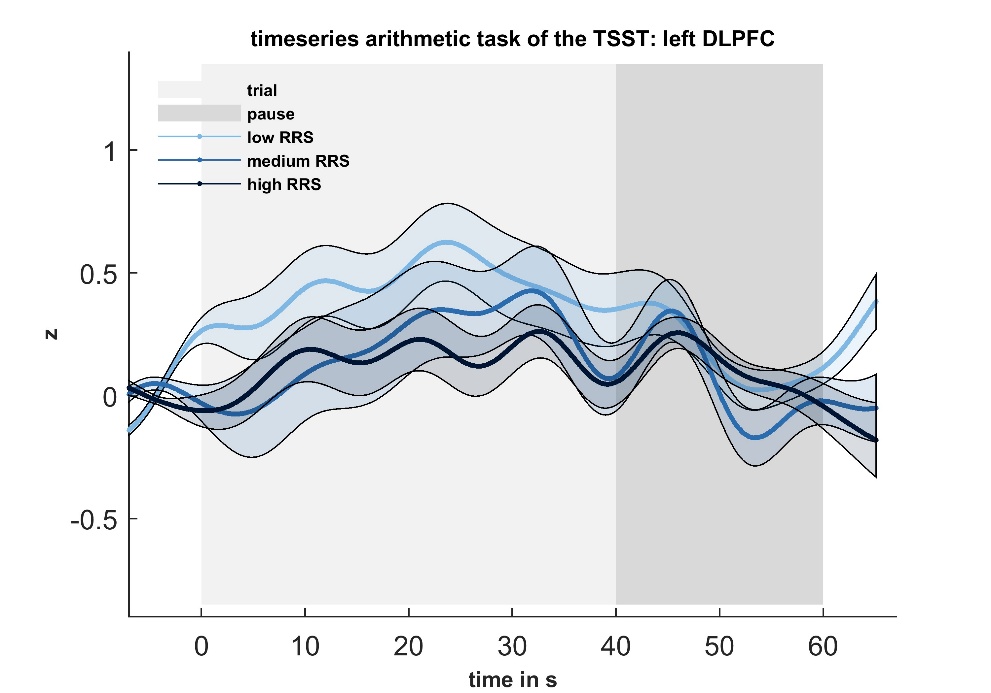

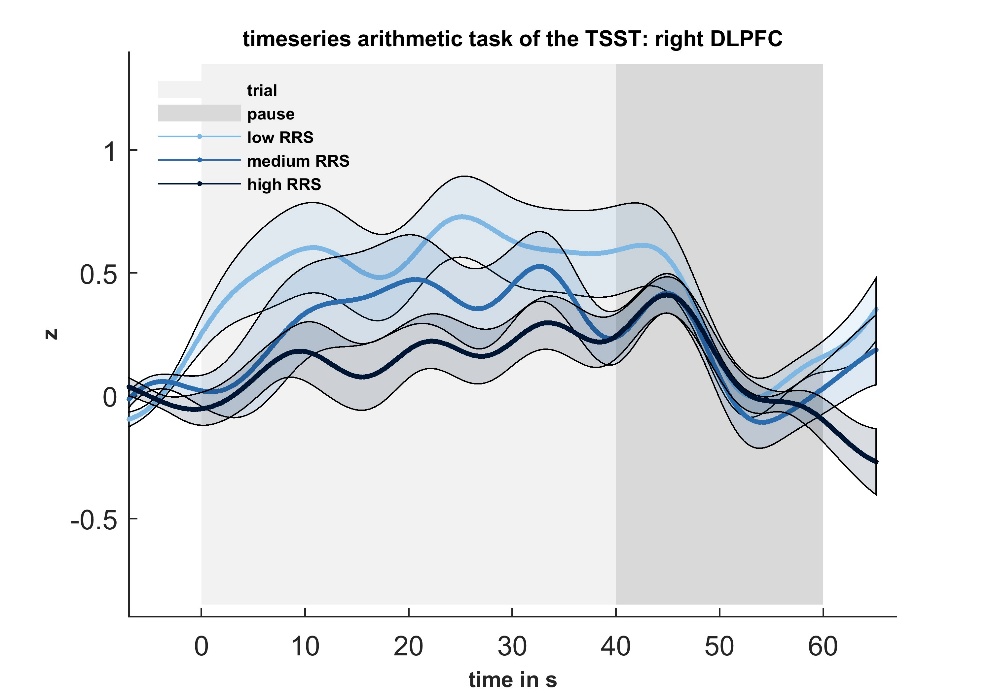

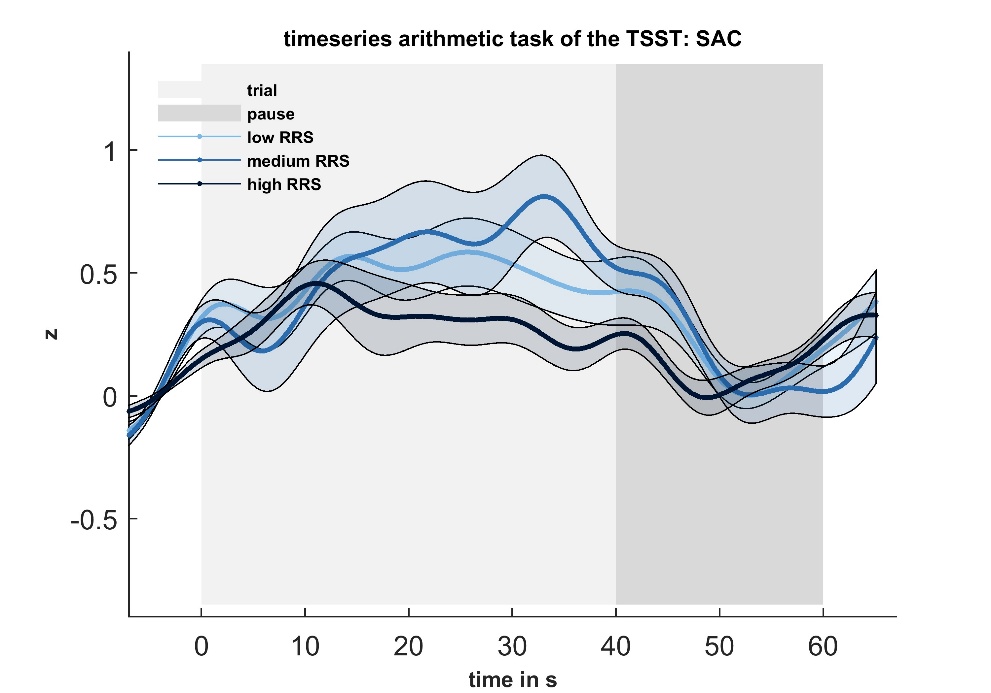


**Supplementary material S15:** Reliable Change Indices in Rosenbaum et al. (2018)

**Table S15** Absolute and relative frequencies of reliable change according to Reliable Change Indices (RCI).

|  | reliable decrease | no reliable change | reliable increase | test-statistic |
| --- | --- | --- | --- | --- |
| low RRS  (*n* = 22) | 0  (0.00%) | 17  (77.27%) | 5  (22.73%) | 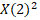= 5.678, *p* = 0.058 |
| high RRS  (*n* = 23) | 1  (4.35%) | 10  (43.48%) | 12  (52.17%) |  |

*Note.* Percentages refer to the relative frequencies in the corresponding subsample (low, medium or high ruminators) and the test-statistic indicated
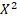
-test comparing the distribution of RCI-categories in the subsamples.


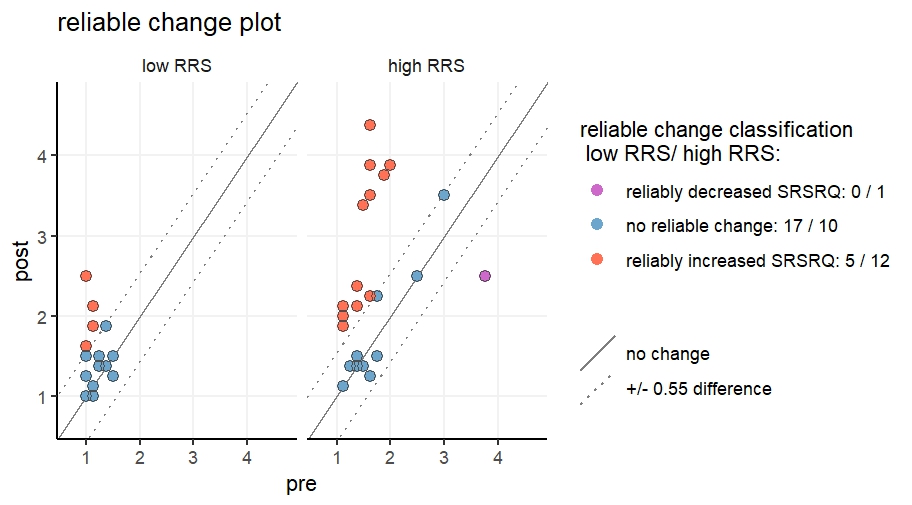


**Figure S15.** Reliable change index plotted for changes in state rumination previous to the stress induction (rest1) to after the stress induction (rest2) dependent on RRS group.

**References**

Bader, K., Hänny, C., Schäfer, V., Neuckel, A., Kuhl, C., 2009. Childhood Trauma Questionnaire – Psychometrische Eigenschaften einer deutschsprachigen Version. Zeitschrift für Klinische Psychologie und Psychotherapie 38, 223–230. https://doi.org/10.1026/1616-3443.38.4.223

Baker, S.L., Heinrichs, N., Kim, H.-J., Hofmann, S.G., 2002. The Liebowitz social anxiety scale as a self-report instrument: a preliminary psychometric analysis. Behaviour Research and Therapy 40, 701–715. https://doi.org/10.1016/S0005-7967(01)00060-2

Beck, A.T., Steer, R.A., Brown, G.K., others, 1996. Beck depression inventory.

Beck, A.T., Ward, C.H., Mendelson, M., Mock, J., Erbaugh, J., 1961. An inventory for measuring depression. Archives of general psychiatry 4, 561–571.

Bernstein, David P., Fink, L., Handelsman, L., Foote, J., 1994. Childhood Trauma Questionnaire. https://doi.org/10.1037/t02080-000

Bernstein, David P, Fink, L., Handelsman, L., Foote, J., Lovejoy, M., Wenzel, K., Sapareto, E., Ruggiero, J., 1994. Initial reliability and validity of a new retrospective measure of child abuse and neglect. The American journal of psychiatry 151, 1132–1136.

Bernstein, D.P., Stein, J.A., Newcomb, M.D., Walker, E., Pogge, D., Ahluvalia, T., Stokes, J., Handelsman, L., Medrano, M., Desmond, D., Zule, W., 2003. Development and validation of a brief screening version of the Childhood Trauma Questionnaire. Child Abuse & Neglect 27, 169–190. https://doi.org/10.1016/S0145-2134(02)00541-0

Burns, E.E., Fischer, S., Jackson, J.L., Harding, H.G., 2012. Deficits in emotion regulation mediate the relationship between childhood abuse and later eating disorder symptoms. Child Abuse & Neglect 36, 32–39. https://doi.org/10.1016/j.chiabu.2011.08.005

Burns, E.E., Jackson, J.L., Harding, H.G., 2010. Child Maltreatment, Emotion Regulation, and Posttraumatic Stress: The Impact of Emotional Abuse. Journal of Aggression, Maltreatment & Trauma 19, 801–819. https://doi.org/10.1080/10926771.2010.522947

Crawford, J.R., Henry, J.D., 2004. The Positive and Negative Affect Schedule (PANAS): Construct validity, measurement properties and normative data in a large non-clinical sample. British Journal of Clinical Psychology 43, 245–265. https://doi.org/10.1348/0144665031752934

Cui, X., Bray, S., Reiss, A.L., 2010. Functional Near Infrared Spectroscopy (NIRS) signal improvement based on negative correlation between oxygenated and deoxygenated hemoglobin dynamics. NeuroImage 49, 3039. https://doi.org/10.1016/j.neuroimage.2009.11.050

Fishburn, F.A., Ludlum, R.S., Vaidya, C.J., Medvedev, A.V., 2019. Temporal Derivative Distribution Repair (TDDR): A motion correction method for fNIRS. NeuroImage 184, 171–179. https://doi.org/10.1016/j.neuroimage.2018.09.025

Hautzinger, M., Keller, F., Kühner, C., 2009. Beck - Depressions - Inventar (BDI-II) Deutsche Ausgabe. Harcourt Test Services GmbH.

Heimberg, R.G., Holaway, R.M., 2007. Examination of the known-groups validity of the Liebowitz Social Anxiety Scale. Depression and Anxiety 24, 447–454. https://doi.org/10.1002/da.20277

Heimberg, R.G., Horner, K.J., Juster, H.R., Safren, S.A., Brown, E.J., Schneier, F.R., Liebowitz, M.R., 1999. Psychometric properties of the Liebowitz Social Anxiety Scale. Psychological Medicine 29, 199–212. https://doi.org/10.1017/S0033291798007879

Huh, H.J., Kim, K.H., Lee, H.-K., Chae, J.-H., 2017. The relationship between childhood trauma and the severity of adulthood depression and anxiety symptoms in a clinical sample: The mediating role of cognitive emotion regulation strategies. Journal of Affective Disorders 213, 44–50. https://doi.org/10.1016/j.jad.2017.02.009

Int-Veen, I., Laicher, H., Ehlis, A.-C., Fallgatter, A.J., Rosenbaum, D., n.d. Measuring state rumination: Development and psychometric evaluation of the stress-reactive state rumination questionnaire (SRSRQ).

Just, N., Alloy, L.B., 1997. The response styles theory of depression: Tests and an extension of the theory. Journal of Abnormal Psychology 106, 221–229. https://doi.org/10.1037/0021-843X.106.2.221

Kasch, K.L., Klein, D.N., Lara, M.E., 2001. A construct validation study of the Response Styles Questionnaire Rumination scale in participants with a recent-onset major depressive episode. Psychological Assessment 13, 375–383. https://doi.org/10.1037/1040-3590.13.3.375

Klinitzke, G., Romppel, M., Häuser, W., Brähler, E., Glaesmer, H., 2012. Die deutsche Version des Childhood Trauma Questionnaire (CTQ) – psychometrische Eigenschaften in einer bevölkerungsrepräsentativen Stichprobe. PPmP - Psychotherapie · Psychosomatik · Medizinische Psychologie 62, 47–51. https://doi.org/10.1055/s-0031-1295495

Krohne, H.W., Egloff, B., Kohlmann, C.-W., Tausch, A., 2016. Positive and Negative Affect Schedule–German Version. American Psychological Association. https://doi.org/10.1037/t49650-000

Kühner, C., Bürger, C., Keller, F., Hautzinger, M., 2007. Reliabilität und Validität des revidierten Beck-Depressionsinventars (BDI-II): Befunde aus deutschsprachigen Stichproben. Der Nervenarzt 78, 651–656. https://doi.org/10.1007/s00115-006-2098-7

Kumar, G., Steer, R.A., Teitelman, K.B., Villacis, L., 2002. Effectiveness of Beck Depression Inventory–Ii Subscales in Screening for Major Depressive Disorders in Adolescent Psychiatric Inpatients. Assessment 9, 164–170. https://doi.org/10.1177/10791102009002007

Liebowitz, M.R., 1987. Social Phobia, in: Klein, D.F. (Ed.), Modern Trends in Pharmacopsychiatry. S. Karger AG, pp. 141–173. https://doi.org/10.1159/000414022

Moberly, N.J., Watkins, E.R., 2008. Ruminative self-focus, negative life events, and negative affect. Behaviour Research and Therapy 46, 1034–1039. https://doi.org/10.1016/j.brat.2008.06.004

Nolen-Hoeksema, S., Larson, J., Grayson, C., 1999. Explaining the gender difference in depressive symptoms. Journal of Personality and Social Psychology 77, 1061–1072. https://doi.org/10.1037/0022-3514.77.5.1061

Nolen-Hoeksema, S., Morrow, J., 1991. A prospective study of depression and posttraumatic stress symptoms after a natural disaster: The 1989 Loma Prieta earthquake. Journal of Personality and Social Psychology 61, 115–121. https://doi.org/10.1037/0022-3514.61.1.115

Nolen-Hoeksema, S., Parker, L.E., Larson, J., 1994. Ruminative coping with depressed mood following loss. Journal of Personality and Social Psychology 67, 92–104. https://doi.org/10.1037/0022-3514.67.1.92

Okamoto, M., Dan, H., Sakamoto, K., Takeo, K., Shimizu, K., Kohno, S., Oda, I., Isobe, S., Suzuki, T., Kohyama, K., others, 2004. Three-dimensional probabilistic anatomical cranio-cerebral correlation via the international 10–20 system oriented for transcranial functional brain mapping. Neuroimage 21, 99–111.

Okamoto, M., Dan, I., 2005. Automated cortical projection of head-surface locations for transcranial functional brain mapping. Neuroimage 26, 18–28.

Rosenbaum, D., Thomas, M., Hilsendegen, P., Metzger, F.G., Haeussinger, F.B., Nuerk, H.-C., Fallgatter, A.J., Nieratschker, V., Ehlis, A.-C., 2018. Stress-related dysfunction of the right inferior frontal cortex in high ruminators: An fNIRS study. NeuroImage: Clinical 18, 510–517. https://doi.org/10.1016/j.nicl.2018.02.022

Rytwinski, N.K., Fresco, D.M., Heimberg, R.G., Coles, M.E., Liebowitz, M.R., Cissell, S., Stein, M.B., Hofmann, S.G., 2009. Screening for social anxiety disorder with the self-report version of the Liebowitz Social Anxiety Scale. Depression and Anxiety 26, 34–38. https://doi.org/10.1002/da.20503

Sassaroli, A., Fantini, S., 2004. Comment on the modified Beer–Lambert law for scattering media. Physics in Medicine & Biology 49, N255.

Scher, C.D., Stein, M.B., Asmundson, G.J.G., McCreary, D.R., Forde, D.R., 2001. The childhood trauma questionnaire in a community sample: Psychometric properties and normative data. Journal of Traumatic Stress 14, 843–857. https://doi.org/10.1023/A:1013058625719

Singh, A.K., Okamoto, M., Dan, H., Jurcak, V., Dan, I., 2005. Spatial registration of multichannel multi-subject fNIRS data to MNI space without MRI. Neuroimage 27, 842–851.

Treynor, W., Gonzalez, R., 2003. Rumination Reconsidered: A Psychometric Analysis. Cognitive Therapy and Research 27. https://doi.org/10.1023/A:1023910315561

von Glischinski, M., von Brachel, R., Hirschfeld, G., 2019. How depressed is “depressed”? A systematic review and diagnostic meta-analysis of optimal cut points for the Beck Depression Inventory revised (BDI-II). Quality of Life Research 28, 1111–1118.

Wahl, K., Ertle, A., Bohne, A., Zurowski, B., Kordon, A., 2011. Relations between a ruminative thinking style and obsessive–compulsive symptoms in non-clinical samples. Anxiety, Stress & Coping 24, 217–225. https://doi.org/10.1080/10615806.2010.482985

Wang, Y.-P., Gorenstein, C., 2013. Psychometric properties of the Beck Depression Inventory-II: a comprehensive review. Brazilian Journal of Psychiatry 35, 416–431.

Watson, D., Clark, L.A., Tellegen, A., 1988. Development and validation of brief measures of positive and negative affect: The PANAS scales. Journal of Personality and Social Psychology 54, 1063–1070. https://doi.org/10.1037/0022-3514.54.6.1063

Wingenfeld, K., Spitzer, C., Mensebach, C., Grabe, H., Hill, A., Gast, U., Schlosser, N., Höpp, H., Beblo, T., Driessen, M., 2010. Die deutsche Version des Childhood Trauma Questionnaire (CTQ): Erste Befunde zu den psychometrischen Kennwerten. PPmP - Psychotherapie · Psychosomatik · Medizinische Psychologie 60, 442–450. https://doi.org/10.1055/s-0030-1247564
