# Supplementary material for: Rumination Out Loud? Linguistic, Neural, and Psychophysiological Correlates of the Think‐Aloud Paradigm
Source: Depress Anxiety. 2026 Apr 23;2026:8855110. doi: 10.1155/da/8855110 (PMC13106887; doi:10.1155/da/8855110)
Supplement: Supplementary file 2 — Supporting Information 2 Syntax_SPSS.doc: provides the SPSS syntax used for the analysis. [file DA-2026-8855110-s001.doc]

* Encoding: UTF-8.

* Analysis Think Aloud TSST

* last edited 2025_06_25 by Isabell Int-Veen

* Encoding: UTF-8.

* open data

GET

FILE=

'Q:\NAS\Ehlis_Auswertung\Laufwerk_S\AG-Mitglieder\David\Artikel\TSST-TAP\2025_06_20_Daten_ThinkAl'+

'oudTSST_nurcompleter_qualiRatingsergänzt.sav'.

DATASET NAME DataSet1 WINDOW=FRONT.

* Benjamini Hochberg

sort cases by p (a).

compute i=$casenum.

sort cases by i (d).

compute q=.05.

compute m=max(i,lag(m)).

compute crit=q*i/m.

compute test=(p le crit).

compute test=max(test,lag(test)).

execute.

formats i m test(f8.0) q (f8.2) crit(f8.6).

value labels test 1 'Significant' 0 'Not Significant'.

* demografische Daten

FREQUENCIES VARIABLES=ID

/FORMAT=NOTABLE

/ORDER=ANALYSIS.

SORT CASES BY Gruppe.

SPLIT FILE LAYERED BY Gruppe.

DESCRIPTIVES VARIABLES=BADO_Alter BDI_totalscore RRS_mean LSAS_totalscore CTQ_totalscore

/STATISTICS=MEAN STDDEV MIN MAX.

FREQUENCIES VARIABLES=BADO_Geschlecht

/ORDER=ANALYSIS.

SPLIT FILE OFF.

UNIANOVA BADO_Alter BY Gruppe

/METHOD=SSTYPE(3)

/INTERCEPT=INCLUDE

/PRINT ETASQ

/CRITERIA=ALPHA(.05)

/DESIGN=Gruppe.

CROSSTABS

/TABLES=BADO_Geschlecht BY Gruppe

/FORMAT=AVALUE TABLES

/STATISTICS=CHISQ

/CELLS=COUNT

/COUNT ROUND CELL.

UNIANOVA BDI_totalscore BY Gruppe

/METHOD=SSTYPE(3)

/INTERCEPT=INCLUDE

/PRINT ETASQ

/CRITERIA=ALPHA(.05)

/DESIGN=Gruppe.

UNIANOVA RRS_mean BY Gruppe

/METHOD=SSTYPE(3)

/INTERCEPT=INCLUDE

/PRINT ETASQ

/CRITERIA=ALPHA(.05)

/DESIGN=Gruppe.

UNIANOVA LSAS_totalscore BY Gruppe

/METHOD=SSTYPE(3)

/INTERCEPT=INCLUDE

/PRINT ETASQ

/CRITERIA=ALPHA(.05)

/DESIGN=Gruppe.

* Mahalanobis distance self-censorship

OUTPUT CLOSE ALL.

OUTPUT NEW.

REGRESSION

/MISSING LISTWISE

/STATISTICS COEFF OUTS R ANOVA

/CRITERIA=PIN(.05) POUT(.10)

/NOORIGIN

/DEPENDENT ID

/METHOD=ENTER TAP_rest1_selfcensored TAP_rest2_selfcensored

/SAVE MAHAL.

COMPUTE Probability_MAH_1=1-CDF.CHISQ(MAH_1,2).

EXECUTE.

FREQUENCIES VARIABLES=ID

/FORMAT=NOTABLE

/ORDER=ANALYSIS.

FILTER OFF.

USE ALL.

SELECT IF (Probability_MAH_1 >= 0.001).

EXECUTE.

* 2 outliers, p < .001: VP70, 61

FREQUENCIES VARIABLES=ID

/FORMAT=NOTABLE

/ORDER=ANALYSIS.

*self-censorship

GLM TAP_rest1_selfcensored TAP_rest2_selfcensored BY Gruppe

/WSFACTOR=time 2 Polynomial

/METHOD=SSTYPE(3)

/PLOT=PROFILE(time*Gruppe) TYPE=LINE ERRORBAR=SE(1) MEANREFERENCE=NO YAXIS=AUTO

/PRINT=DESCRIPTIVE ETASQ OPOWER

/CRITERIA=ALPHA(.05)

/WSDESIGN=time

/DESIGN=Gruppe.

* Mahalanobis distance subjective stress

OUTPUT CLOSE ALL.

OUTPUT NEW.

GET

FILE=

'Q:\NAS\Ehlis_Auswertung\Laufwerk_S\AG-Mitglieder\David\Artikel\TSST-TAP\2025_06_20_Daten_ThinkAl'+

'oudTSST_nurcompleter_qualiRatingsergänzt.sav'.

DATASET NAME DataSet1 WINDOW=FRONT.

REGRESSION

/MISSING LISTWISE

/STATISTICS COEFF OUTS R ANOVA

/CRITERIA=PIN(.05) POUT(.10)

/NOORIGIN

/DEPENDENT ID

/METHOD=ENTER VAS_1 VAS_2 VAS_3 VAS_4 VAS_5 VAS_6 VAS_7 VAS_8

/SAVE MAHAL.

COMPUTE Probability_MAH_1=1-CDF.CHISQ(MAH_1,8).

EXECUTE.

FREQUENCIES VARIABLES=ID

/FORMAT=NOTABLE

/ORDER=ANALYSIS.

FILTER OFF.

USE ALL.

SELECT IF (Probability_MAH_1 >= 0.001).

EXECUTE.

* 0 outliers p < .001

FREQUENCIES VARIABLES=ID

/FORMAT=NOTABLE

/ORDER=ANALYSIS.

GLM VAS_1 VAS_2 VAS_3 VAS_4 VAS_5 VAS_6 VAS_7 VAS_8 BY Gruppe

/WSFACTOR=time 8 Polynomial

/METHOD=SSTYPE(3)

/PLOT=PROFILE(time*Gruppe) TYPE=LINE ERRORBAR=SE(1) MEANREFERENCE=NO YAXIS=AUTO

/EMMEANS=TABLES(Gruppe) COMPARE ADJ(LSD)

/EMMEANS=TABLES(time) COMPARE ADJ(LSD)

/PRINT=DESCRIPTIVE ETASQ OPOWER

/CRITERIA=ALPHA(.05)

/WSDESIGN=time

/DESIGN=Gruppe.

* Mahalanobis distance state rumination

OUTPUT CLOSE ALL.

OUTPUT NEW.

GET

FILE=

'Q:\NAS\Ehlis_Auswertung\Laufwerk_S\AG-Mitglieder\David\Artikel\TSST-TAP\2025_06_20_Daten_ThinkAl'+

'oudTSST_nurcompleter_qualiRatingsergänzt.sav'.

DATASET NAME DataSet1 WINDOW=FRONT.

REGRESSION

/MISSING LISTWISE

/STATISTICS COEFF OUTS R ANOVA

/CRITERIA=PIN(.05) POUT(.10)

/NOORIGIN

/DEPENDENT ID

/METHOD=ENTER staterum1_totalscore staterum2_totalscore

/SAVE MAHAL.

COMPUTE Probability_MAH_1=1-CDF.CHISQ(MAH_1,2).

EXECUTE.

FREQUENCIES VARIABLES=ID

/FORMAT=NOTABLE

/ORDER=ANALYSIS.

FILTER OFF.

USE ALL.

SELECT IF (Probability_MAH_1 >= 0.001).

EXECUTE.

* 1 outlier, p < .001: VP48

FREQUENCIES VARIABLES=ID

/FORMAT=NOTABLE

/ORDER=ANALYSIS.

GLM staterum1_totalscore staterum2_totalscore BY Gruppe

/WSFACTOR=time 2 Polynomial

/METHOD=SSTYPE(3)

/PLOT=PROFILE(time*Gruppe) TYPE=LINE ERRORBAR=SE(1) MEANREFERENCE=NO YAXIS=AUTO

/EMMEANS=TABLES(Gruppe) COMPARE ADJ(LSD)

/PRINT=DESCRIPTIVE ETASQ OPOWER

/CRITERIA=ALPHA(.05)

/WSDESIGN=time

/DESIGN=Gruppe.

* Chi-Quadrat Test state rumination RCI

CROSSTABS

/TABLES=Gruppe BY RCI

/FORMAT=AVALUE TABLES

/STATISTICS=CHISQ

/CELLS=COUNT

/COUNT ROUND CELL.

* Mahalanobis distance positive affect

OUTPUT CLOSE ALL.

OUTPUT NEW.

GET

FILE=

'Q:\NAS\Ehlis_Auswertung\Laufwerk_S\AG-Mitglieder\David\Artikel\TSST-TAP\2025_06_20_Daten_ThinkAl'+

'oudTSST_nurcompleter_qualiRatingsergänzt.sav'.

DATASET NAME DataSet1 WINDOW=FRONT.

REGRESSION

/MISSING LISTWISE

/STATISTICS COEFF OUTS R ANOVA

/CRITERIA=PIN(.05) POUT(.10)

/NOORIGIN

/DEPENDENT ID

/METHOD=ENTER PANAS1_PA PANAS2_PA

/SAVE MAHAL.

COMPUTE Probability_MAH_1=1-CDF.CHISQ(MAH_1,2).

EXECUTE.

FREQUENCIES VARIABLES=ID

/FORMAT=NOTABLE

/ORDER=ANALYSIS.

FILTER OFF.

USE ALL.

SELECT IF (Probability_MAH_1 >= 0.001).

EXECUTE.

* 0 outlier, p < .001

FREQUENCIES VARIABLES=ID

/FORMAT=NOTABLE

/ORDER=ANALYSIS.

GLM PANAS1_PAmean PANAS2_PAmean BY Gruppe

/WSFACTOR=time 2 Polynomial

/METHOD=SSTYPE(3)

/PLOT=PROFILE(time*Gruppe) TYPE=LINE ERRORBAR=SE(1) MEANREFERENCE=NO YAXIS=AUTO

/EMMEANS=TABLES(Gruppe) COMPARE ADJ(LSD)

/PRINT=DESCRIPTIVE ETASQ OPOWER

/CRITERIA=ALPHA(.05)

/WSDESIGN=time

/DESIGN=Gruppe.

* PANAS negative affect

* Mahalanobis distance negative affect

OUTPUT CLOSE ALL.

OUTPUT NEW.

GET

FILE=

'Q:\NAS\Ehlis_Auswertung\Laufwerk_S\AG-Mitglieder\David\Artikel\TSST-TAP\2025_06_20_Daten_ThinkAl'+

'oudTSST_nurcompleter_qualiRatingsergänzt.sav'.

DATASET NAME DataSet1 WINDOW=FRONT.

REGRESSION

/MISSING LISTWISE

/STATISTICS COEFF OUTS R ANOVA

/CRITERIA=PIN(.05) POUT(.10)

/NOORIGIN

/DEPENDENT ID

/METHOD=ENTER PANAS1_NA PANAS2_NA

/SAVE MAHAL.

COMPUTE Probability_MAH_1=1-CDF.CHISQ(MAH_1,2).

EXECUTE.

FREQUENCIES VARIABLES=ID

/FORMAT=NOTABLE

/ORDER=ANALYSIS.

FILTER OFF.

USE ALL.

SELECT IF (Probability_MAH_1 >= 0.001).

EXECUTE.

* 0 outlier, p < .001

FREQUENCIES VARIABLES=ID

/FORMAT=NOTABLE

/ORDER=ANALYSIS.

GLM PANAS1_NAmean PANAS2_NAmean BY Gruppe

/WSFACTOR=time 2 Polynomial

/METHOD=SSTYPE(3)

/PLOT=PROFILE(time*Gruppe) TYPE=LINE ERRORBAR=SE(1) MEANREFERENCE=NO YAXIS=AUTO

/EMMEANS=TABLES(Gruppe) COMPARE ADJ(LSD)

/PRINT=DESCRIPTIVE ETASQ OPOWER

/CRITERIA=ALPHA(.05)

/WSDESIGN=time

/DESIGN=Gruppe.

* Mahalanobis distance nuber of performed calculations

OUTPUT CLOSE ALL.

OUTPUT NEW.

GET

FILE=

'Q:\NAS\Ehlis_Auswertung\Laufwerk_S\AG-Mitglieder\David\Artikel\TSST-TAP\2025_06_20_Daten_ThinkAl'+

'oudTSST_nurcompleter_qualiRatingsergänzt.sav'.

DATASET NAME DataSet1 WINDOW=FRONT.

REGRESSION

/MISSING LISTWISE

/STATISTICS COEFF OUTS R ANOVA

/CRITERIA=PIN(.05) POUT(.10)

/NOORIGIN

/DEPENDENT ID

/METHOD=ENTER TSST_SummeCTRL1 TSST_SummeCTRL2 TSST_SummeTSST

/SAVE MAHAL.

COMPUTE Probability_MAH_1=1-CDF.CHISQ(MAH_1,3).

EXECUTE.

FREQUENCIES VARIABLES=ID

/FORMAT=NOTABLE

/ORDER=ANALYSIS.

FILTER OFF.

USE ALL.

SELECT IF (Probability_MAH_1 >= 0.001).

EXECUTE.

* 0 outlier, p < .001

FREQUENCIES VARIABLES=ID

/FORMAT=NOTABLE

/ORDER=ANALYSIS.

GLM TSST_SummeCTRL1 TSST_SummeCTRL2 TSST_SummeTSST BY Gruppe

/WSFACTOR=time 3 Polynomial

/METHOD=SSTYPE(3)

/PLOT=PROFILE(time*Gruppe) TYPE=LINE ERRORBAR=SE(1) MEANREFERENCE=NO YAXIS=AUTO

/EMMEANS=TABLES(Gruppe) COMPARE ADJ(LSD)

/EMMEANS=TABLES(time) COMPARE ADJ(LSD)

/PRINT=DESCRIPTIVE ETASQ OPOWER

/CRITERIA=ALPHA(.05)

/WSDESIGN=time

/DESIGN=Gruppe.

* Mahalanobis distance nuber of errors

OUTPUT CLOSE ALL.

OUTPUT NEW.

GET

FILE=

'Q:\NAS\Ehlis_Auswertung\Laufwerk_S\AG-Mitglieder\David\Artikel\TSST-TAP\2025_06_20_Daten_ThinkAl'+

'oudTSST_nurcompleter_qualiRatingsergänzt.sav'.

DATASET NAME DataSet1 WINDOW=FRONT.

REGRESSION

/MISSING LISTWISE

/STATISTICS COEFF OUTS R ANOVA

/CRITERIA=PIN(.05) POUT(.10)

/NOORIGIN

/DEPENDENT ID

/METHOD=ENTER TSST_FehlerCTRL1 TSST_FehlerCTRL2 TSST_FehlerTSST

/SAVE MAHAL.

COMPUTE Probability_MAH_1=1-CDF.CHISQ(MAH_1,3).

EXECUTE.

FREQUENCIES VARIABLES=ID

/FORMAT=NOTABLE

/ORDER=ANALYSIS.

FILTER OFF.

USE ALL.

SELECT IF (Probability_MAH_1 >= 0.001).

EXECUTE.

* 0 outlier, p < .001

FREQUENCIES VARIABLES=ID

/FORMAT=NOTABLE

/ORDER=ANALYSIS.

GLM TSST_FehlerCTRL1 TSST_FehlerCTRL2 TSST_FehlerTSST BY Gruppe

/WSFACTOR=time 3 Polynomial

/METHOD=SSTYPE(3)

/PLOT=PROFILE(time*Gruppe) TYPE=LINE ERRORBAR=SE(1) MEANREFERENCE=NO YAXIS=AUTO

/EMMEANS=TABLES(Gruppe) COMPARE ADJ(LSD)

/EMMEANS=TABLES(time) COMPARE ADJ(LSD)

/PRINT=DESCRIPTIVE ETASQ OPOWER

/CRITERIA=ALPHA(.05)

/WSDESIGN=time

/DESIGN=Gruppe.

* heart rate

* no multivariate outlier, missing data: n = 13

OUTPUT CLOSE ALL.

OUTPUT NEW.

GET

FILE=

'Q:\NAS\Ehlis_Auswertung\Laufwerk_S\AG-Mitglieder\David\Artikel\TSST-TAP\2025_06_20_Daten_ThinkAl'+

'oudTSST_nurcompleter_qualiRatingsergänzt.sav'.

DATASET NAME DataSet1 WINDOW=FRONT.

REGRESSION

/MISSING LISTWISE

/STATISTICS COEFF OUTS R ANOVA

/CRITERIA=PIN(.05) POUT(.10)

/NOORIGIN

/DEPENDENT ID

/METHOD=ENTER BPM_rest1 BPM_ctrl1 BPM_ctrl2 BPM_anti BPM_speech BPM_arith BPM_rest2

/SAVE MAHAL.

COMPUTE Probability_MAH_1=1-CDF.CHISQ(MAH_1,7).

EXECUTE.

FREQUENCIES VARIABLES=ID

/FORMAT=NOTABLE

/ORDER=ANALYSIS.

FILTER OFF.

USE ALL.

SELECT IF (Probability_MAH_1 >= 0.001).

EXECUTE.

* 0 outlier, p < .001

FREQUENCIES VARIABLES=ID

/FORMAT=NOTABLE

/ORDER=ANALYSIS.

GLM BPM_rest1 BPM_ctrl1 BPM_ctrl2 BPM_anti BPM_speech BPM_arith BPM_rest2 BY Gruppe

/WSFACTOR=time 7 Polynomial

/METHOD=SSTYPE(3)

/PLOT=PROFILE(time*Gruppe) TYPE=LINE ERRORBAR=SE(1) MEANREFERENCE=NO YAXIS=AUTO

/EMMEANS=TABLES(time) COMPARE ADJ(LSD)

/PRINT=DESCRIPTIVE ETASQ OPOWER

/CRITERIA=ALPHA(.05)

/WSDESIGN=time

/DESIGN=Gruppe.

* Mahalanobis distance fNIRS MANOVA und rmANOVA

OUTPUT CLOSE ALL.

OUTPUT NEW.

GET

FILE=

'Q:\NAS\Ehlis_Auswertung\Laufwerk_S\AG-Mitglieder\David\Artikel\TSST-TAP\2025_06_20_Daten_ThinkAl'+

'oudTSST_nurcompleter_qualiRatingsergänzt.sav'.

DATASET NAME DataSet1 WINDOW=FRONT.

REGRESSION

/MISSING LISTWISE

/STATISTICS COEFF OUTS R ANOVA

/CRITERIA=PIN(.05) POUT(.10)

/NOORIGIN

/DEPENDENT ID

/METHOD=ENTER CTL1_lDLPFC_Cui CTL2_lDLPFC_Cui Arith_lDLPFC_Cui CTL1_lIFG_Cui CTL2_lIFG_Cui Arith_lIFG_Cui

CTL1_rDLPFC_Cui CTL2_rDLPFC_Cui Arith_rDLPFC_Cui CTL1_rIFG_Cui CTL2_rIFG_Cui Arith_rIFG_Cui

CTL1_SAC_Cui CTL2_SAC_Cui Arith_SAC_Cui

/SAVE MAHAL.

COMPUTE Probability_MAH_1=1-CDF.CHISQ(MAH_1,15).

EXECUTE.

FREQUENCIES VARIABLES=ID

/FORMAT=NOTABLE

/ORDER=ANALYSIS.

FILTER OFF.

USE ALL.

SELECT IF (Probability_MAH_1 >= 0.001).

EXECUTE.

* 0 outliers, p < .001; missing data: n = 1 (VP35)

FREQUENCIES VARIABLES=ID

/FORMAT=NOTABLE

/ORDER=ANALYSIS.

GLM CTL1_lIFG_Cui CTL2_lIFG_Cui Arith_lIFG_Cui CTL1_rIFG_Cui CTL2_rIFG_Cui Arith_rIFG_Cui

CTL1_lDLPFC_Cui CTL2_lDLPFC_Cui Arith_lDLPFC_Cui CTL1_rDLPFC_Cui CTL2_rDLPFC_Cui Arith_rDLPFC_Cui

CTL1_SAC_Cui CTL2_SAC_Cui Arith_SAC_Cui BY Gruppe

/WSFACTOR=ROI 5 Polynomial time 3 Polynomial

/CONTRAST(Gruppe)=Helmert

/METHOD=SSTYPE(3)

/PLOT=PROFILE(time*Gruppe time*ROI time*Gruppe*ROI) TYPE=LINE ERRORBAR=SE(1) MEANREFERENCE=NO

YAXIS=AUTO

/EMMEANS=TABLES(Gruppe*time) COMPARE(Gruppe) ADJ(LSD)

/EMMEANS=TABLES(Gruppe*time) COMPARE(time) ADJ(LSD)

/EMMEANS=TABLES(ROI*time) COMPARE(ROI) ADJ(LSD)

/EMMEANS=TABLES(ROI*time) COMPARE(time) ADJ(LSD)

/PRINT=DESCRIPTIVE ETASQ

/CRITERIA=ALPHA(.05)

/WSDESIGN=ROI time ROI*time

/DESIGN=Gruppe.

* Mahalanobis distance Sentiment-ANOVA

OUTPUT CLOSE ALL.

OUTPUT NEW.

GET

FILE=

'Q:\NAS\Ehlis_Auswertung\Laufwerk_S\AG-Mitglieder\David\Artikel\TSST-TAP\2025_06_20_Daten_ThinkAl'+

'oudTSST_nurcompleter_qualiRatingsergänzt.sav'.

DATASET NAME DataSet1 WINDOW=FRONT.

REGRESSION

/MISSING LISTWISE

/STATISTICS COEFF OUTS R ANOVA

/CRITERIA=PIN(.05) POUT(.10)

/NOORIGIN

/DEPENDENT ID

/METHOD=ENTER SentimentScore_rest1 SentimentScore_rest2

/SAVE MAHAL.

COMPUTE Probability_MAH_1=1-CDF.CHISQ(MAH_1,2).

EXECUTE.

FREQUENCIES VARIABLES=ID

/FORMAT=NOTABLE

/ORDER=ANALYSIS.

FILTER OFF.

USE ALL.

SELECT IF (Probability_MAH_1 >= 0.001).

EXECUTE.

* 0 outliers, p < .001

FREQUENCIES VARIABLES=ID

/FORMAT=NOTABLE

/ORDER=ANALYSIS.

* Sentiment-Analysis

DATASET ACTIVATE DataSet1.

GLM SentimentScore_rest1 SentimentScore_rest2 BY Gruppe

/WSFACTOR=time 2 Polynomial

/METHOD=SSTYPE(3)

/PLOT=PROFILE(time*Gruppe) TYPE=LINE ERRORBAR=SE(1) MEANREFERENCE=NO YAXIS=AUTO

/EMMEANS=TABLES(Gruppe) COMPARE ADJ(LSD)

/PRINT=DESCRIPTIVE ETASQ

/CRITERIA=ALPHA(.05)

/WSDESIGN=time

/DESIGN=Gruppe.

* Mahalanobis distance qualitative analyse (observer-rated state rumination): scale 1

OUTPUT CLOSE ALL.

OUTPUT NEW.

GET

FILE=

'Q:\NAS\Ehlis_Auswertung\Laufwerk_S\AG-Mitglieder\David\Artikel\TSST-TAP\2025_06_20_Daten_ThinkAl'+

'oudTSST_nurcompleter_qualiRatingsergänzt.sav'.

DATASET NAME DataSet1 WINDOW=FRONT.

REGRESSION

/MISSING LISTWISE

/STATISTICS COEFF OUTS R ANOVA

/CRITERIA=PIN(.05) POUT(.10)

/NOORIGIN

/DEPENDENT ID

/METHOD=ENTER quali_rehashing_rest1 quali_rehashing_rest2

/SAVE MAHAL.

COMPUTE Probability_MAH_1=1-CDF.CHISQ(MAH_1,2).

EXECUTE.

FREQUENCIES VARIABLES=ID

/FORMAT=NOTABLE

/ORDER=ANALYSIS.

FILTER OFF.

USE ALL.

SELECT IF (Probability_MAH_1 >= 0.001).

EXECUTE.

FREQUENCIES VARIABLES=ID

/FORMAT=NOTABLE

/ORDER=ANALYSIS.

DATASET ACTIVATE DataSet1.

GLM quali_rehashing_rest1 quali_rehashing_rest2 BY Gruppe

/WSFACTOR=time 2 Polynomial

/METHOD=SSTYPE(3)

/PLOT=PROFILE(time*Gruppe) TYPE=LINE ERRORBAR=SE(1) MEANREFERENCE=NO YAXIS=AUTO

/PRINT=DESCRIPTIVE ETASQ

/CRITERIA=ALPHA(.05)

/WSDESIGN=time

/DESIGN=Gruppe.

* Mahalanobis distance qualitative analyse (observer-rated state rumination): scale 2

OUTPUT CLOSE ALL.

OUTPUT NEW.

GET

FILE=

'Q:\NAS\Ehlis_Auswertung\Laufwerk_S\AG-Mitglieder\David\Artikel\TSST-TAP\2025_06_20_Daten_ThinkAl'+

'oudTSST_nurcompleter_qualiRatingsergänzt.sav'.

DATASET NAME DataSet1 WINDOW=FRONT.

REGRESSION

/MISSING LISTWISE

/STATISTICS COEFF OUTS R ANOVA

/CRITERIA=PIN(.05) POUT(.10)

/NOORIGIN

/DEPENDENT ID

/METHOD=ENTER quali_consequences_rest1 quali_consequences_rest2

/SAVE MAHAL.

COMPUTE Probability_MAH_1=1-CDF.CHISQ(MAH_1,2).

EXECUTE.

FREQUENCIES VARIABLES=ID

/FORMAT=NOTABLE

/ORDER=ANALYSIS.

FILTER OFF.

USE ALL.

SELECT IF (Probability_MAH_1 >= 0.001).

EXECUTE.

FREQUENCIES VARIABLES=ID

/FORMAT=NOTABLE

/ORDER=ANALYSIS.

DATASET ACTIVATE DataSet1.

GLM quali_consequences_rest1 quali_consequences_rest2 BY Gruppe

/WSFACTOR=time 2 Polynomial

/METHOD=SSTYPE(3)

/PLOT=PROFILE(time*Gruppe) TYPE=LINE ERRORBAR=SE(1) MEANREFERENCE=NO YAXIS=AUTO

/PRINT=DESCRIPTIVE ETASQ

/CRITERIA=ALPHA(.05)

/WSDESIGN=time

/DESIGN=Gruppe.

* Mahalanobis distance qualitative analyse (observer-rated state rumination): scale 3

OUTPUT CLOSE ALL.

OUTPUT NEW.

GET

FILE=

'Q:\NAS\Ehlis_Auswertung\Laufwerk_S\AG-Mitglieder\David\Artikel\TSST-TAP\2025_06_20_Daten_ThinkAl'+

'oudTSST_nurcompleter_qualiRatingsergänzt.sav'.

DATASET NAME DataSet1 WINDOW=FRONT.

REGRESSION

/MISSING LISTWISE

/STATISTICS COEFF OUTS R ANOVA

/CRITERIA=PIN(.05) POUT(.10)

/NOORIGIN

/DEPENDENT ID

/METHOD=ENTER quali_affect_rest1 quali_affect_rest2

/SAVE MAHAL.

COMPUTE Probability_MAH_1=1-CDF.CHISQ(MAH_1,2).

EXECUTE.

FREQUENCIES VARIABLES=ID

/FORMAT=NOTABLE

/ORDER=ANALYSIS.

FILTER OFF.

USE ALL.

SELECT IF (Probability_MAH_1 >= 0.001).

EXECUTE.

FREQUENCIES VARIABLES=ID

/FORMAT=NOTABLE

/ORDER=ANALYSIS.

GLM quali_affect_rest1 quali_affect_rest2 BY Gruppe

/WSFACTOR=time 2 Polynomial

/METHOD=SSTYPE(3)

/PLOT=PROFILE(time*Gruppe) TYPE=LINE ERRORBAR=SE(1) MEANREFERENCE=NO YAXIS=AUTO

/EMMEANS=TABLES(Gruppe) COMPARE ADJ(LSD)

/PRINT=DESCRIPTIVE ETASQ

/CRITERIA=ALPHA(.05)

/WSDESIGN=time

/DESIGN=Gruppe.

* Mahalanobis distance qualitative analyse (observer-rated state rumination): scale 4

OUTPUT CLOSE ALL.

OUTPUT NEW.

GET

FILE=

'Q:\NAS\Ehlis_Auswertung\Laufwerk_S\AG-Mitglieder\David\Artikel\TSST-TAP\2025_06_20_Daten_ThinkAl'+

'oudTSST_nurcompleter_qualiRatingsergänzt.sav'.

DATASET NAME DataSet1 WINDOW=FRONT.

REGRESSION

/MISSING LISTWISE

/STATISTICS COEFF OUTS R ANOVA

/CRITERIA=PIN(.05) POUT(.10)

/NOORIGIN

/DEPENDENT ID

/METHOD=ENTER quali_reflection_rest1 quali_reflection_rest2

/SAVE MAHAL.

COMPUTE Probability_MAH_1=1-CDF.CHISQ(MAH_1,2).

EXECUTE.

FREQUENCIES VARIABLES=ID

/FORMAT=NOTABLE

/ORDER=ANALYSIS.

FILTER OFF.

USE ALL.

SELECT IF (Probability_MAH_1 >= 0.001).

EXECUTE.

FREQUENCIES VARIABLES=ID

/FORMAT=NOTABLE

/ORDER=ANALYSIS.

GLM quali_reflection_rest1 quali_reflection_rest2 BY Gruppe

/WSFACTOR=time 2 Polynomial

/METHOD=SSTYPE(3)

/PLOT=PROFILE(time*Gruppe) TYPE=LINE ERRORBAR=SE(1) MEANREFERENCE=NO YAXIS=AUTO

/EMMEANS=TABLES(Gruppe) COMPARE ADJ(LSD)

/PRINT=DESCRIPTIVE ETASQ

/CRITERIA=ALPHA(.05)

/WSDESIGN=time

/DESIGN=Gruppe.

DATASET CLOSE DataSet1.

REGRESSION

/MISSING LISTWISE

/STATISTICS COEFF OUTS R ANOVA

/CRITERIA=PIN(.05) POUT(.10)

/NOORIGIN

/DEPENDENT ID

/METHOD=ENTER staterum1_totalscore staterum2_totalscore

/SAVE MAHAL.

COMPUTE Probability_MAH_1=1-CDF.CHISQ(MAH_1,2).

EXECUTE.

FREQUENCIES VARIABLES=ID

/FORMAT=NOTABLE

/ORDER=ANALYSIS.

FILTER OFF.

USE ALL.

SELECT IF (Probability_MAH_1 >= 0.001).

EXECUTE.

* 1 outlier, p < .001: VP48

FREQUENCIES VARIABLES=ID

/FORMAT=NOTABLE

/ORDER=ANALYSIS.

CORRELATIONS

/VARIABLES=SentimentScore_Differenz staterum_Differenz NA_Differenz

/PRINT=ONETAIL NOSIG FULL

/MISSING=PAIRWISE.

CORRELATIONS

/VARIABLES=SentimentScore_rest1 staterum1_totalscore PANAS1_NAmean

/PRINT=ONETAIL NOSIG FULL

/MISSING=PAIRWISE.

CORRELATIONS

/VARIABLES=SentimentScore_rest2 staterum2_totalscore PANAS2_NAmean

/PRINT=ONETAIL NOSIG FULL

/MISSING=PAIRWISE.

CORRELATIONS

/VARIABLES=SentimentScore_Differenz NIRS_lIFG_DifferenzTSSTCTL1 NIRS_rIFG_DifferenzTSSTCTL1

NIRS_lDLPFC_DifferenzTSSTCTL1 NIRS_rDLPFC_DifferenzTSSTCTL1 NIRS_SAC_DifferenzTSSTCTL1

/PRINT=ONETAIL NOSIG LNODIAG

/MISSING=PAIRWISE.

CORRELATIONS

/VARIABLES=quali_rehashing_rest1 quali_consequences_rest1 quali_affect_rest1

quali_reflection_rest1 staterum1_totalscore staterum2_totalscore

/PRINT=ONETAIL NOSIG LNODIAG

/MISSING=PAIRWISE.

CORRELATIONS

/VARIABLES=staterum_changescore quali_rehashing_changescore quali_consequences_changescore

quali_affect_changescore quali_reflection_changescore

/PRINT=ONETAIL NOSIG LNODIAG

/MISSING=PAIRWISE.

* fNIRS Helmert-Kontraste der Interaktion time*group

DATASET ACTIVATE DataSet1.

GLM CTL1_lIFG_Cui CTL2_lIFG_Cui Arith_lIFG_Cui CTL1_rIFG_Cui CTL2_rIFG_Cui Arith_rIFG_Cui

CTL1_lDLPFC_Cui CTL2_lDLPFC_Cui Arith_lDLPFC_Cui CTL1_rDLPFC_Cui CTL2_rDLPFC_Cui Arith_rDLPFC_Cui

CTL1_SAC_Cui CTL2_SAC_Cui Arith_SAC_Cui BY Helmertgroup_1

/WSFACTOR=ROI 5 Polynomial time 3 Polynomial

/METHOD=SSTYPE(3)

/PRINT=DESCRIPTIVE ETASQ

/CRITERIA=ALPHA(.05)

/WSDESIGN=ROI time ROI*time

/DESIGN=Helmertgroup_1.

GLM CTL1_lIFG_Cui CTL2_lIFG_Cui Arith_lIFG_Cui CTL1_rIFG_Cui CTL2_rIFG_Cui Arith_rIFG_Cui

CTL1_lDLPFC_Cui CTL2_lDLPFC_Cui Arith_lDLPFC_Cui CTL1_rDLPFC_Cui CTL2_rDLPFC_Cui Arith_rDLPFC_Cui

CTL1_SAC_Cui CTL2_SAC_Cui Arith_SAC_Cui BY Helmertgroup_2

/WSFACTOR=ROI 5 Polynomial time 3 Polynomial

/METHOD=SSTYPE(3)

/PRINT=DESCRIPTIVE ETASQ

/CRITERIA=ALPHA(.05)

/WSDESIGN=ROI time ROI*time

/DESIGN=Helmertgroup_2.

* fNIRS post-hoc tests der Interaktion time*ROI (separate rmANOVAs je ROI rechnen und den linearen Kontrast des Faktors Zeit bereichten)

DATASET ACTIVATE DataSet1.

GLM CTL1_lIFG_Cui CTL2_lIFG_Cui Arith_lIFG_Cui BY Gruppe

/WSFACTOR=time 3 Polynomial

/METHOD=SSTYPE(3)

/PLOT=PROFILE(time) TYPE=LINE ERRORBAR=SE(1) MEANREFERENCE=NO YAXIS=AUTO

/PRINT=DESCRIPTIVE ETASQ

/CRITERIA=ALPHA(.05)

/WSDESIGN=time

/DESIGN=Gruppe.

GLM CTL1_rIFG_Cui CTL2_rIFG_Cui Arith_rIFG_Cui BY Gruppe

/WSFACTOR=time 3 Polynomial

/METHOD=SSTYPE(3)

/PLOT=PROFILE(time) TYPE=LINE ERRORBAR=SE(1) MEANREFERENCE=NO YAXIS=AUTO

/PRINT=DESCRIPTIVE ETASQ

/CRITERIA=ALPHA(.05)

/WSDESIGN=time

/DESIGN=Gruppe.

GLM CTL1_lDLPFC_Cui CTL2_lDLPFC_Cui Arith_lDLPFC_Cui BY Gruppe

/WSFACTOR=time 3 Polynomial

/METHOD=SSTYPE(3)

/PLOT=PROFILE(time) TYPE=LINE ERRORBAR=SE(1) MEANREFERENCE=NO YAXIS=AUTO

/PRINT=DESCRIPTIVE ETASQ

/CRITERIA=ALPHA(.05)

/WSDESIGN=time

/DESIGN=Gruppe.

GLM CTL1_rDLPFC_Cui CTL2_rDLPFC_Cui Arith_rDLPFC_Cui BY Gruppe

/WSFACTOR=time 3 Polynomial

/METHOD=SSTYPE(3)

/PLOT=PROFILE(time) TYPE=LINE ERRORBAR=SE(1) MEANREFERENCE=NO YAXIS=AUTO

/PRINT=DESCRIPTIVE ETASQ

/CRITERIA=ALPHA(.05)

/WSDESIGN=time

/DESIGN=Gruppe.

GLM CTL1_SAC_Cui CTL2_SAC_Cui Arith_SAC_Cui BY Gruppe

/WSFACTOR=time 3 Polynomial

/METHOD=SSTYPE(3)

/PLOT=PROFILE(time) TYPE=LINE ERRORBAR=SE(1) MEANREFERENCE=NO YAXIS=AUTO

/PRINT=DESCRIPTIVE ETASQ

/CRITERIA=ALPHA(.05)

/WSDESIGN=time

/DESIGN=Gruppe.
